# Superior efficacy of cotreatment with BET protein inhibitor and BCL2 or MCL1 inhibitor against AML blast progenitor cells

**DOI:** 10.1038/s41408-018-0165-5

**Published:** 2019-01-15

**Authors:** Warren Fiskus, Tianyu Cai, Courtney D. DiNardo, Steven M. Kornblau, Gautam Borthakur, Tapan M. Kadia, Naveen Pemmaraju, Prithviraj Bose, Lucia Masarova, Kimal Rajapakshe, Dimuthu Perera, Cristian Coarfa, Christopher P. Mill, Dyana T. Saenz, David N. Saenz, Baohua Sun, Joseph D. Khoury, Yu Shen, Marina Konopleva, Kapil N. Bhalla

**Affiliations:** 1 The University of Texas MD Anderson Cancer Center, Houston, TX 77030 USA; 2 Department of Molecular and Cellular Biology, Baylor College of Medicine, Houston, TX 77030 USA; 3 AbbVie, Inc., North Chicago, IL 60064 USA

## Abstract

First-generation bromodomain extra-terminal protein (BETP) inhibitors (BETi) (e.g., OTX015) that disrupt binding of BETP BRD4 to chromatin transcriptionally attenuate AML-relevant progrowth and prosurvival oncoproteins. BETi treatment induces apoptosis of AML BPCs, reduces in vivo AML burden and induces clinical remissions in a minority of AML patients. Clinical efficacy of more potent BETis, e.g., ABBV-075 (AbbVie, Inc.), is being evaluated. Venetoclax and A-1210477 bind and inhibit the antiapoptotic activity of BCL2 and MCL1, respectively, lowering the threshold for apoptosis. BETi treatment is shown here to perturb accessible chromatin and activity of enhancers/promoters, attenuating MYC, CDK6, MCL1 and BCL2, while inducing BIM, HEXIM1, CDKN1A expressions and apoptosis of AML cells. Treatment with venetoclax increased MCL1 protein levels, but cotreatment with ABBV-075 reduced MCL1 and Bcl-xL levels. ABBV-075 cotreatment synergistically induced apoptosis with venetoclax or A-1210477 in patient-derived, CD34+ AML cells. Compared to treatment with either agent alone, cotreatment with ABBV-075 and venetoclax was significantly more effective in reducing AML cell-burden and improving survival, without inducing toxicity, in AML-engrafted immune-depleted mice. These findings highlight the basis of superior activity and support interrogation of clinical efficacy and safety of cotreatment with BETi and BCL2 or MCL1 inhibitor in AML.

## Introduction

The bromodomain extra-terminal (BET) protein (BETP) BRD4 interacts with transcription factors as well as cofactors, including mediator protein complex, lysine methyltransferase NSD3, arginine demethylase JMJD6, and pTEFb (a heterodimer of CDK9 and cyclin T), to regulate RNA pol II (RNAP2)-mediated transcript elongation^1–4^. BRD4 promotes pTEFb-mediated phosphorylation of serine 2 in the heptad repeats within the CTD of RNAP2, as well as of the negative transcription elongation factors, NELF and Sept5, which induces promoter-proximal pause release of RNAP2 and RNA transcript elongation^4–6^. This has been shown to occur at the enhancers and promoters of oncogenes that promote growth and survival of cancer cells, including acute myeloid leukemia (AML) stem-progenitor cells^2,6–9^. Consistent with this, knockdown of BRD4 by RNAi, or disruption of its binding to acetylated chromatin by BET inhibitors (BETi) leads to lethality in AML blast progenitor cells (BPCs), associated with down regulation of AML-relevant progrowth and prosurvival oncogenes^1,2,10–13^. BETis, including JQ1 and OTX015, have been documented to reduce AML burden and improve survival of mice engrafted with human AML BPCs^11–13^. Whereas treatment with BETi was shown to induce clinical responses in AML, refractoriness to BETi therapy and resistance with disease progression is uniformly observed^14–16^. This has prompted the development and testing of more potent and effective BETis, e.g., ABBV-075^16–20^. Since BETi treatment attenuated expressions of several BCL2 family of antiapoptotic proteins^11–13,21^, to further lower the threshold for apoptosis and enhance clinical anti-AML efficacy of BETi, a logical approach would be to concomitantly target and inhibit activity of the antiapoptotic proteins.

BCL2, Bcl-xL, and MCL1 are members of multi-BCL-2 homology (BH) domain (BH1−BH4) containing family of antiapoptotic proteins^22,23^. They bind proapoptotic BCL2 family members BAX and BAK (containing BH1, BH2, and BH3) and BH3 domain-only proapoptotic activator proteins, to inhibit intrinsic mitochondria-induced pathway of apoptosis^22–24^. The first, highly selective BCL2 inhibitor venetoclax (ABT-199) binds specifically to BCL2 and displaces BH3 domain-only proteins to trigger BAX/BAK-mediated mitochondria-induced apoptosis of cancer, including AML cells^25,26^. Venetoclax treatment alone showed anti-AML in vivo efficacy in the mouse xenograft models^26,27^. Although effective in inducing clinical remissions in AML, innate or acquired resistance to venetoclax alone is commonly observed^28^. The best predictor of sustained response to venetoclax is the lack of readily accessible resistance mechanisms provided by Bcl-xL and MCL1^28^. In venetoclax-resistant cells, increased MCL1 and/or Bcl-xL levels was observed^29^. Preclinically, dual targeting of BCL2 and MCL1, but not either alone, was also shown to prolong survival of AML or lymphoma bearing mice^30,31^. Combining venetoclax with other anti-AML drugs such as cytarabine or DNA hypomethylating agent has yielded higher remission rates^32,33^. However, a full assessment of their clinical efficacy has not been conducted. In present studies we determined the effects of the BETi on *cis*-regulatory DNA elements and on mRNA and protein expressions of AML-relevant oncoproteins, including MYC, BCL2, Bcl-xL, MCL1, and CDK6. We also determined whether the BETi ABBV-075-induced perturbations in protein levels of these oncoproteins are associated with synergistic in vitro activity of ABBV-075 and venetoclax or the MCL1 inhibitor A-1210477. Finally, we also determined whether combination of ABBV-075 and venetoclax exerted superior in vivo efficacy against mouse xenograft models of AML BPCs.

## Materials and methods

### Reagents and antibodies

In vivo grade ABBV-075 and ABT-199 for mouse xenograft experiments were kindly provided by AbbVie, Inc. In vitro grade A-1210477 (Catalog No. S7790), ABBV-075 (Catalog No. S8400) and ABT-199 (Catalog No. S8048) were purchased from Selleck Chemicals (Houston, TX) and utilized for in vitro experiments. All compounds were prepared as 10 mM stocks in 100% dimethyl sulfoxide (DMSO) and frozen at −80 °C in 5–10 µL aliquots to allow for single use, thus avoiding multiple freeze-thaw cycles that could result in compound decomposition and loss of activity. Anti-BRD4 (RRID:AB_2620184) antibody was obtained from Bethyl Labs (Montgomery, TX). Anti-c-Myc (RRID: AB_1903938), anti-HEXIM1 (Cat #12604), anti-p21 (RRID: AB_823586), anti-p-Histone H2AX (Ser139) (RRID: AB_2118010), anti-Bcl-xL (RRID: AB_10695729), anti-BAX (RRID:AB_2744530), anti-BAK (RRID:AB_2290287), anti-Cleaved PARP (RRID:AB_331426), anti-BIM (RRID:AB_1030947) and anti-MCL1 (RRID:AB_2281980) antibodies were obtained from Cell Signaling Technologies (Beverly, MA). Anti-CDK6 (RRID: AB_10610066), anti-Bcl2 (RRID: AB_626733), Alexa488-conjugated anti-BAX(6A7) (RRID:AB_626728) and anti-β-Actin (RRID: AB_626630) antibodies were obtained from Santa Cruz Biotechnologies (Santa Cruz, CA). Anti-BAK(NT) (RRID:AB_310159) antibody was obtained from Millipore Sigma (Burlington, MA).

### Cell lines and cell culture

Human AML cell lines MOLM13 (RRID:CVCL_2119), SET2 (RRID:CVCL_2187), SKM1 (RRID:CVCL_0098) and OCI-AML5 (RRID:CVCL_1620) were obtained from the DSMZ (Braunschweig, Germany). MV4-11 cells were obtained from the ATCC (Manassas, VA). All experiments with cell lines were performed within 6 months after thawing or obtaining from ATCC or DSMZ. The cell lines were also authenticated in the Characterized Cell Line Core Facility at M.D. Anderson Cancer Center, Houston TX. SKM1 and MOLM13 cells were cultured in RPMI media with 20% fetal bovine serum (FBS) and 1% penicillin/streptomycin and 1% non-essential amino acids. OCI-AML5 cells were cultured in MEM alpha media with 20% FBS and 1% penicillin/streptomycin and 1% non-essential amino acids. OCI-AML5 cultures were also supplemented with 10 ng/mL of GM-CSF (PeproTech, Rocky Hill, NJ). MV4-11 cells were cultured in ATCC-formulated Iscove's Modified Dulbecco's Medium (IMDM) with 20% FBS 1% penicillin/streptomycin and 1% non-essential amino acids. Luciferase-expressing MOLM13 cells were created by transducing cells with Luc-ZSGreen(GFP). pHIV-Luc-ZsGreen was a gift from Bryan Welm (Addgene plasmid #39196). Logarithmically growing, mycoplasma-negative cells were utilized for all experiments. Following drug treatments, cells were washed free of the drug(s) prior to the performance of the studies described.

### Primary AML blast cells

Patient-derived AML cells samples were obtained with informed consent as part of a clinical protocol approved by the Institutional Review Board of The University of Texas, M.D. Anderson Cancer Center. Normal hematopoietic progenitor cells (HPCs) were obtained from delinked, de-identified cord blood samples. Mononuclear cells were purified by Ficoll Hypaque density centrifugation. Mononuclear cells were washed with complete RPMI media containing 20% FBS. CD34+ AML BPCs and normal HPCs were purified by immunomagnetic beads conjugated with anti-CD34 antibody (StemCell Technologies, Vancouver, British Columbia) prior to utilization in the cell viability assays and immunoblot analyses.

### RNA isolation and quantitative-polymerase chain reaction

Following the designated treatments with ABBV-075, total RNA was isolated from AML cells utilizing a PureLink RNA Mini kit from Ambion, Inc. (Austin, TX; RRID: SCR_008406) and reverse transcribed. Quantitative real-time PCR analysis for the expression of *MYC*, *BCL-2*, *BCL2L1 (Bcl-xL)*, *CDK6*, *HEXIM1*, and *CDKN1A (p21*) was performed on cDNA using TaqMan probes from Applied Biosystems (Foster City, CA; RRID: SCR_005039). Relative mRNA expression was normalized to the expression of Glyceraldehyde 3-phosphate dehydrogenase (*GAPDH*) and compared to the untreated cells.

### SDS-PAGE and immunoblot analyses

Seventy five micrograms of total cell lysate were used for SDS-PAGE. Western blot analyses were performed on total cell lysates using specific antisera or monoclonal antibodies. Blots were washed with 1× PBST, then incubated in IRDye 680RD goat anti-mouse (RRID:AB_10956588) or IRDye 800CW goat anti-rabbit (RRID:AB_621843) secondary antibodies (LI-COR, Lincoln, NE) for 1 h, washed three times in 1× Phosphate Buffered Saline with Tween®20 (PBST) and scanned with an Odyssey CLX Infrared Imaging System utilizing Image Studio 5.0 Software (RRID:SCR_015795) (LI-COR, Lincoln, NE). The expression levels of β-Actin in the cell lysates were used as the loading control for the western blots. Immunoblot analyses were performed at least twice. Representative immunoblot images were subjected to densitometry analysis.

### Assessment of apoptosis by annexin V staining

Untreated or drug-treated cells were stained with Annexin V-FITC (Pharmingen, San Diego, CA) and TO-PRO-3 iodide (Life Technologies, Carlsbad, CA) and the percentages of apoptotic cells were determined by flow cytometry. To analyze in vitro synergism between ABBV-075 and ABT-199 or A-1210477 or synergy between A-1210477 and ABT-199, cells were treated with in vitro grade single agents and combinations for 48 h and the percentages of annexin V-positive, apoptotic cells were determined by flow cytometry. The combination index (CI) for each drug combination was calculated by median dose effect and isobologram analyses (assuming mutual exclusivity) utilizing the commercially available software Compusyn. CI values of less than 1.0 represent a synergistic interaction of the two drugs in the combination. The CI values were input into GraphPad V7.0 to create Box and Whisker plots of the range of the CI values for each cell line and drug combination.

### Assessment of percentage nonviable cells

Following designated single agent or combination treatments, primary, patient-derived **(**PD)-AML cells were stained with trypan blue (Sigma, St. Louis, MO). The numbers of nonviable cells were determined by counting the cells that exhibited trypan blue uptake in a hemocytometer, and were reported as a percentage of the untreated control cells. Alternatively, cells were washed with 1× PBS, stained with TO-PRO-3 iodide and analyzed by flow cytometry on an Accuri CFlow6 flow cytometer.

### AML xenograft models

All animal studies were performed under a protocol approved by the IACUC at M.D. Anderson Cancer Center, an AAALAC-accredited institution. To determine the in vivo effects of ABT-199 and/or ABBV-075 on leukemia progression and engraftment, 2 million MOLM13/GFP-Luc cells were injected with a 26-gauge needle into the lateral tail vein of 4–6-week-old female NOD-scid IL2Rgamma^null^ (NSG, Stock number 005557 (RRID:IMSR_JAX:005557); The Jackson Laboratory, Bar Harbor, ME) mice (*n* = 7) which had received a preconditioning dose of radiation (2.5 Gy) 24 h prior to injection of cells. All the mice were monitored for 4 days and imaged to document engraftment. Mice were randomly assigned to treatment cohorts. Following this, mice were treated daily with vehicle (10% ethanol, 30% PEG400, 60% Phosal 50), 50 mg/kg of ABT-199 (by oral gavage, daily × 5 days per week), 1 mg/kg of ABBV-075 (by oral gavage, daily × 5 days per week) or ABBV-075 + ABT-199 for 2 weeks. Mice were injected with 75 mg/kg of d-Luciferin and imaged once per week by Xenogen camera to monitor disease status and treatment efficacy. Mice that became moribund or experienced hind limb paralysis were euthanized according to the approved IACUC protocol. Investigators were not blinded to the experimental conditions; however, veterinarians and veterinary staff assisting in determining when euthanasia was required were blinded to the experimental conditions of the study. The survival of the mice is represented by a Kaplan-Meier plot. The variance between cohorts was similar. To determine the antileukemia effects of ABBV-075 and/or ABT-199 against human AML patient-derived xenograft (PDX) models, NSG mice (*n* = 8 per cohort) were injected with patient-derived AML blast cells following a preconditioning dose of radiation. Mice were monitored for engraftment by flow cytometry. Upon engraftment, mice were treated with ABBV-075 and/or ABT-199 for 22 days. At the end of the treatment period, mice were humanely euthanized and the % of engraftment was documented in the mice.

### Statistical analysis

Significant differences between values obtained in a population of AML cells treated with different experimental conditions were determined using the Student’s *t* test. For the in vivo mouse models, a two-tailed *t* test or a Mantel–Cox Rank sum test was utilized for group comparisons. *p* values of <0.05 were assigned significance.

## Results

### BETi-mediated effects on the gene-regulatory elements and gene-expressions in AML cells

**Fig. 1:**
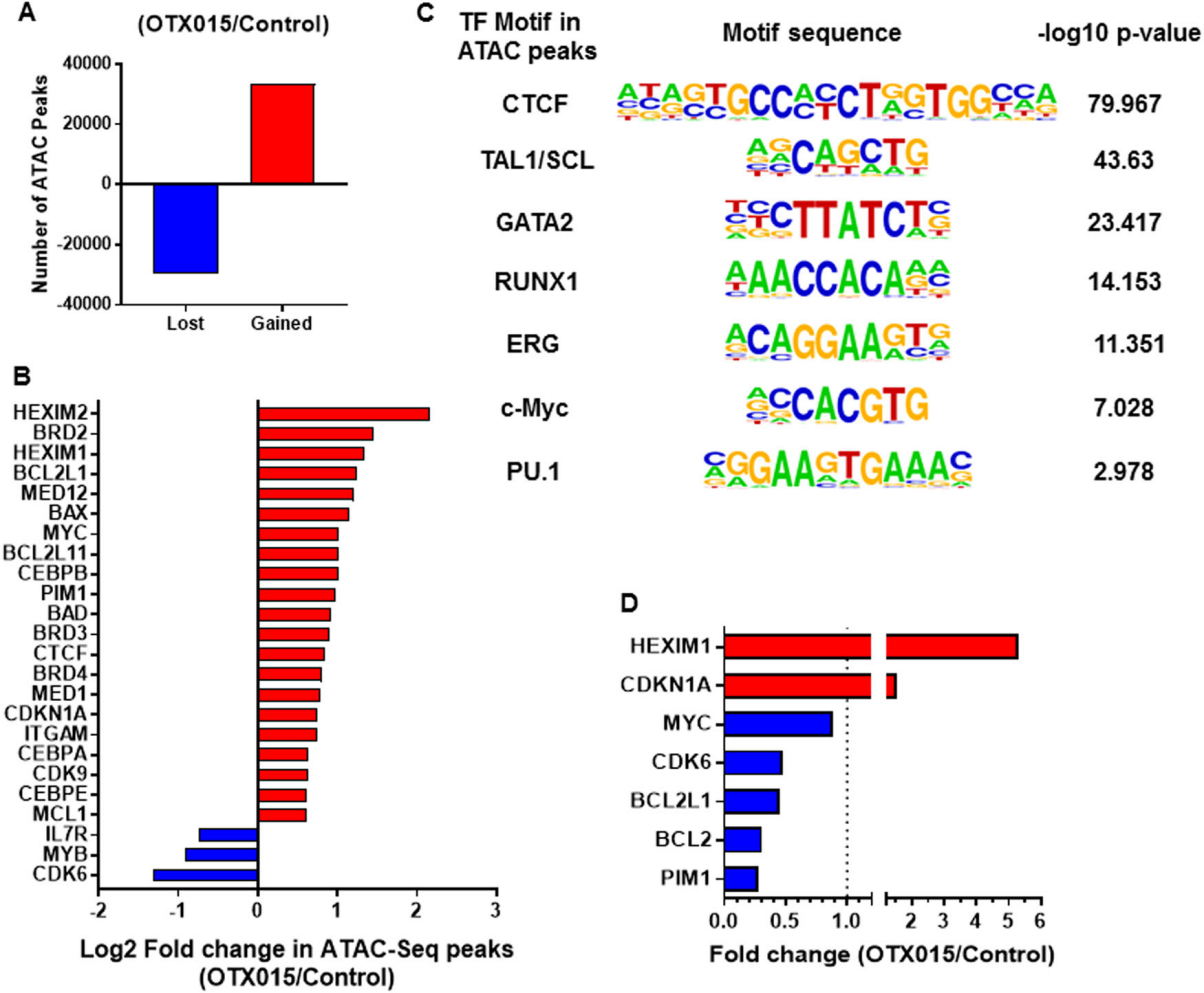
Treatment with BET inhibitor alters chromatin accessibility and mRNA expression levels in AML cells. **a** AML SET2 cells were treated with BET inhibitor OTX015 for 16 h. Cells were harvested and ATAC-Seq analysis was performed. The total number of peaks gained or lost in OTX015-treated cells compared to untreated cells are shown. **b** Log2 fold-change in gained or lost peaks at the transcription start site or within 10 kb of the gene body of leukemia-relevant genes. **c** Transcription factor motifs increased in up (gained) peaks in OTX015-treated AML SET2 cells over control cells and the associated –log10 *p* value. **d** Relative fold-change in BET inhibitor-treated AML SET2 cells over control as determined by RNA-Seq analysis

We first determined the effects of BETi treatment on the open and accessible chromatin, at enhancers and promoters, for transcriptional complexes in AML cells, utilizing ATAC-Seq analysis. Figure 1a, panel a demonstrates large numbers of lost and gained peaks in the chromatin of the AML SET2 cells treated with the BETi OTX015 over untreated SET2 cells. This indicated that BETi treatment markedly affected the accessibility of chromatin to transcriptional complexes. Figure 1b shows log2-fold-change in the ATAC-Seq peaks mapped to transcription start sites ± 10 kb in the DNA of the indicated genes. Notably, BETi treatment increased chromatin accessibility at the promoters and other *cis*-regulatory regions of genes, including *MYC, Bcl-xL (BCL2L1), MCL1, BIM (BCL2L11), HEXIM1/2, PIM1, BAD, CDKN1A, ITGAM (CD11b)*, and *CEBPα/β*, while reducing chromatin accessibility at *cis*-regulatory regions of other genes, including IL7 receptor *(IL7R), MYB*, and *CDK6* (Fig. 1b). In BETi-treated SET2 cells, ATAC-Seq-determined accessible chromatin demonstrated significant enrichment for the canonical binding sites of CTCF, TAL1/SCL, GATA2, RUNX1, ERG, c-Myc, and PU.1 (Fig. 1c). We also determined the status of H3K4Me3 and H3K27Ac marks at the promoter of *MCL1* and *Bcl-xL* in OCI-AML5 or MV4-11 cells, utilizing ChIP-QPCR analyses. Treatment with ABBV-075 did not significantly alter the levels of these chromatin marks at the promoter of *MCL1* or *Bcl-xL* (Fig. S1).

**Fig. 2:**
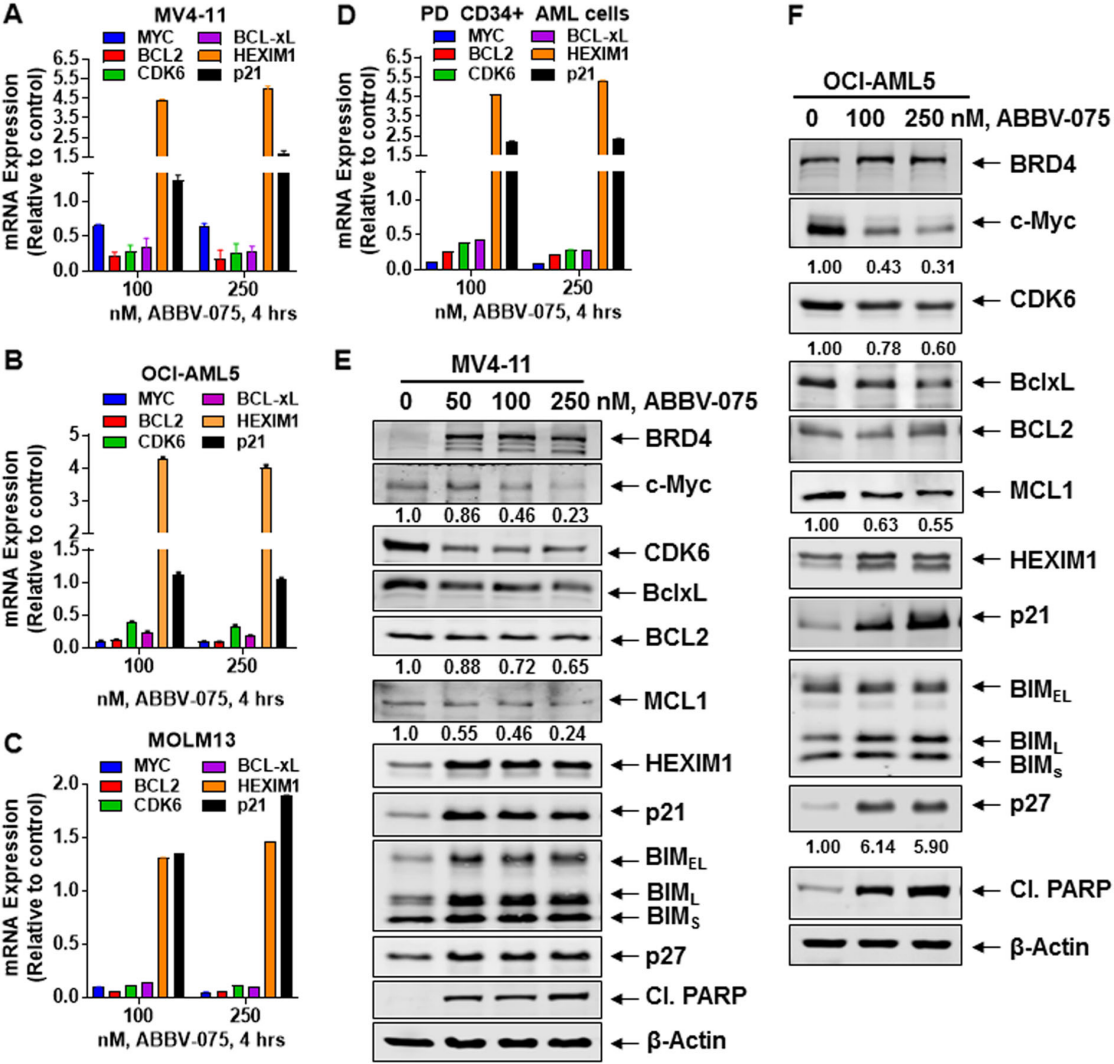
Treatment with the BET inhibitor ABBV-075 depletes expression of super-enhancer regulated genes in AML cells. **a**–**d** MV4-11, OCI-AML5, MOLM13, and primary CD34+ AML cells were treated with the indicated concentrations of ABBV-075 for 4 h. Total RNA was isolated and reverse transcribed. The resulting cDNA was utilized for qPCR. The relative expression of each mRNA was normalized to *GAPDH* and compared to untreated cells (set at 1.0). **e**, **f** MV4-11 and OCI-AML5 cells were treated with the indicated concentrations of ABBV-075 for 24 h. Immunoblot analyses were conducted. The expression levels of β-Actin in the cell lysates served as the loading control. The numbers beneath the bands represent densitometry analysis

Utilizing RNA-Seq analysis, we also determined the impact of BETi-induced perturbations in accessible chromatin on mRNA expressions in the AML SET2 cells. Figure 1d shows the up- or down-regulated mRNA expressions in BETi-treated vs. untreated AML cells. Whereas *HEXIM1* and *CDKN1A (p21)* mRNA levels were induced, mRNA levels of *MYC, CDK6, BCL2L1 (Bcl-xL), BCL2*, and *PIM1* were downregulated. We next confirmed by qPCR analysis whether the more potent BETi ABBV-075 induces similar mRNA perturbations in AML cells, including patient-derived (PD) CD34+ AML BPCs. As shown in Fig. 2a−d, ABBV-075 treatment attenuated *MYC, BCL2, Bcl-xL*, and *CDK6*, while inducing *HEXIM1* and *p21* mRNA levels in MV4-11, OCI-AML5, MOLM13, and PD CD34+ AML BPCs. Genetic alterations detected by NextGen sequencing (NGS) of an AML-associated 28-gene panel, conducted in the AML cell lines, is presented in Fig. S2A. Utilizing a reversed phase protein array (RPPA) and Western analyses, we next determined the effect of ABBV-075 on protein expressions in AML BPCs. Figure S2B demonstrates the heat map of perturbations in protein levels by the RPPA analysis, showing increase in 29 and reduction in 55 protein expressions, following treatment of MV4-11 cells with ABBV-075 for 16 h. As shown in Fig. S2C, among the log2-fold-altered protein levels, proteins involved in cell signaling, cell cycle, and transcription regulators were inhibited, while protein expressions involved in DNA damage response, cell cycle arrest, and cell death were increased. Western analyses confirmed that treatment with ABBV-075 attenuated protein expressions of c-Myc, CDK6, BCL2, and MCL1, while simultaneously inducing the levels of BRD4, HEXIM1, p21, p27, BIM and cleaved PARP in MV4-11, OCI-AML5, and MOLM13 cells (Fig. 2e, f and S2D). Notably, ABBV-075 treatment significantly reduced the expression of MCL1 in MV4-11 and OCI-AML5 AML cells (Fig. S3A and S3B) (*p* < 0.05). Following ABBV-075 treatment, similar effects on the protein levels were observed in three samples of PD CD34+ AML BPCs, including a decline in the levels of MCL1 (Fig. S3C to S3F). We also determined total and activated protein levels of BAX and BAK in AML cells. Whereas the total protein levels of BAX and BAK were unaffected (by Western analyses) (Fig. S4A), activated BAK levels in MOLM13 and activated BAX levels in OCI-AML5 cells were increased (Fig. S4B), following treatment with ABBV-075, as detected by flow cytometry after staining with antibodies that specifically detect the active conformation of BAK or BAX.

### ABBV-075 induces lethal effects in cultured AML cells lines and patient-derived AML blast progenitor cells

We next determined the lethal effects of ABBV-075 in cultured AML cell lines and PD CD34+ AML BPCs. As shown in Fig. S4C and S4D, treatment with ABBV-075 dose-dependently induced apoptosis to a diverse extent in cultured AML cell lines, including FLT3-ITD and MLL fusion-protein expressing MV4-11 and MOLM13 cells. ABBV-075 treatment also induced apoptosis in OCI-AML5, SKM1 and to a lesser extent in Mono-Mac-1 cells (Fig. S4D). Treatment with ABBV-075 also dose-dependently induced lethality in 15 samples of PD CD34+ AML BPCs (seven untreated and eight treatment-refractory) (Figs. S4E and S4F). Specific genetic alterations detected through NGS in these 15 AML samples is shown in Fig. S4G. No notable association was detected between any specific genetic alteration and ABBV-075-mediated loss-of-viability in the PD AML BPCs (Fig. S4E and S4F).

### Venetoclax (ABT-199) and A-1210477 increase MCL1 expression but induce apoptosis in AML cells

**Fig. 3:**
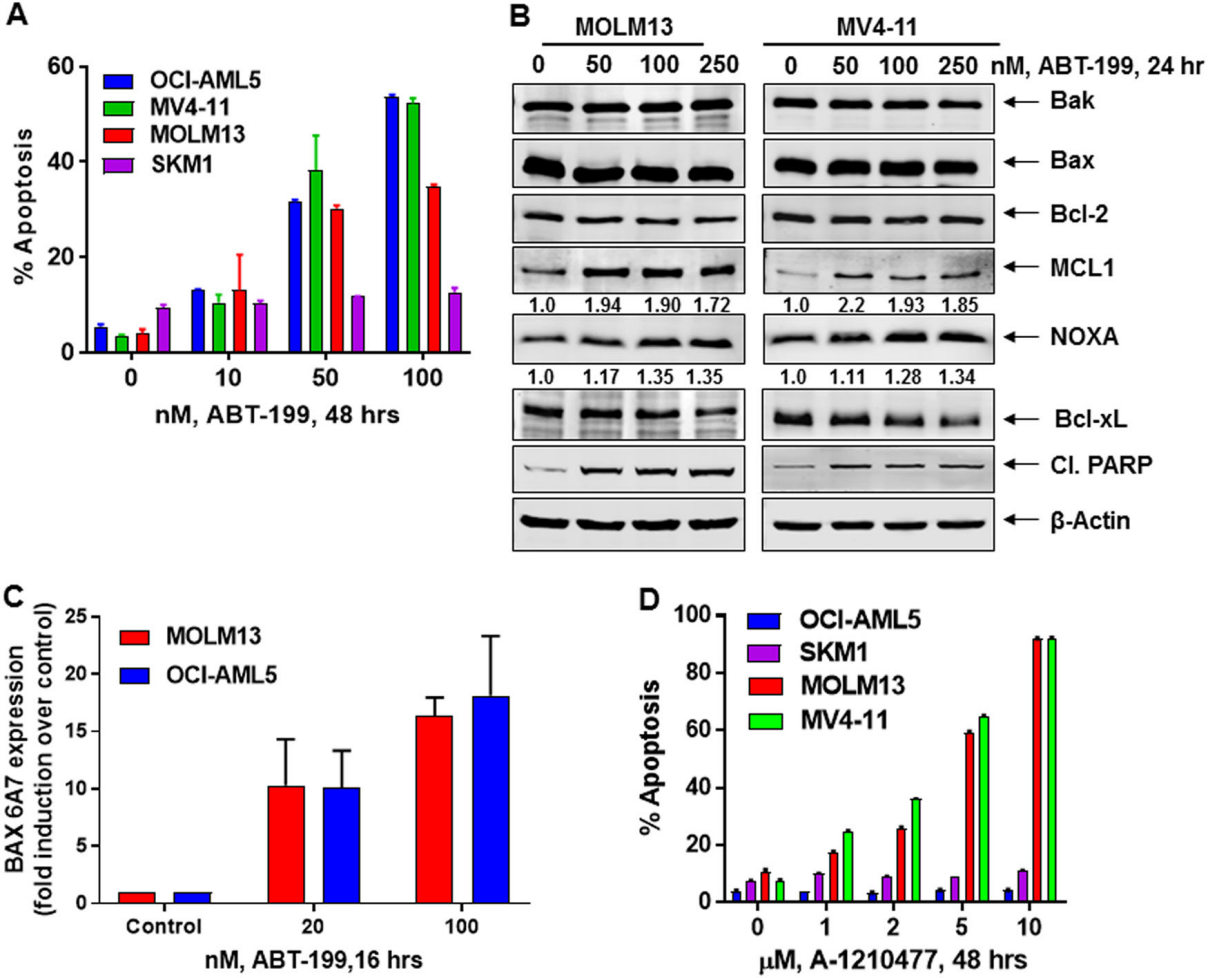
Treatment with ABT-199 (venetoclax) or MCL1 inhibitor A-1210477 induces apoptosis of AML cells. **a** OCI-AML5, MV4-11, MOLM13, and SKM1 cells were treated with the indicated concentrations of ABT-199 for 48 h. Then, the % of annexin V-positive, apoptotic cells were determined by flow cytometry. Columns, mean of three experiments; Bars, Standard error of the mean. **b** MOLM13 and MV4-11 cells were treated with the indicated concentrations of ABT-199 for 24 h. Total cell lysates were prepared and immunoblot analysis was conducted. The expression levels of β-Actin in the cell lysates served as the loading control. The numbers beneath the bands represent densitometry analysis. **c** MOLM13 and OCI-AML5 cells were treated with the indicated concentrations of ABT-199 for 16 h. Cells were fixed, permeabilized and stained with Alexa488-conjugated BAX (6A7) antibody followed by flow cytometry. Values represent fold induction of active BAX over control cells. **d** OCI-AML5, MV4-11, MOLM13, and SKM1 cells were treated with the indicated concentrations of the MCL1-specific inhibitor, A-1210477 for 48 h. The % of annexin V-positive, apoptotic cells were determined by flow cytometry. Columns, mean of three experiments; Bars, Standard error of the mean

We first determined the apoptotic effects of venetoclax alone in the cultured AML cell lines. As shown in Fig. 3a, venetoclax dose-dependently induced apoptosis in OCI-AML5, MV4-11, and MOLM13 but not in SKM1 cells. Although venetoclax did not alter BCL2, BAX, and BAK levels, it did increase activated BAX and BAK levels in MOLM13 and OCI-AML5 cells (Fig. 3b, c and S5A). Notably, venetoclax treatment significantly increased MCL1 levels in these AML cell types (Fig. 3b, S5B and S5C). However, venetoclax simultaneously decreased Bcl-xL levels, while increasing NOXA levels, which has been shown to counter the antiapoptotic activity of MCL1^22–24^ (Fig. 3b and S5B). We next determined the activity of the MCL1 inhibitor A-1210477 against AML BPCs. As shown in Fig. 3d, exposure to A-1210477 dose-dependently induced apoptosis of MOLM13 and MV4-11, but not of OCI-AML5 and SKM1 cells. As previously reported, treatment with A-1210477 increased MCL1 expression by stabilizing MCL1 (Fig. S5D and S5E)^23,34,35^. However, A-1210477 treatment also reduced Bcl-xL and increased active BAK levels in MOLM13 and MV4-11 cells, without a similar effect in OCI-AML5 and SKM1 cells (Fig. S5D-S5F, S5H, and data not shown), which may partially explain A-1210477-induced apoptosis in MOLM13 and MV4-11 but not in OCI-AML5 and SKM1 cells (Fig. 3d).

### Cotreatment with ABBV-075 and venetoclax or A-1210477 synergistically induces in vitro lethality in AML cells

**Fig. 4:**
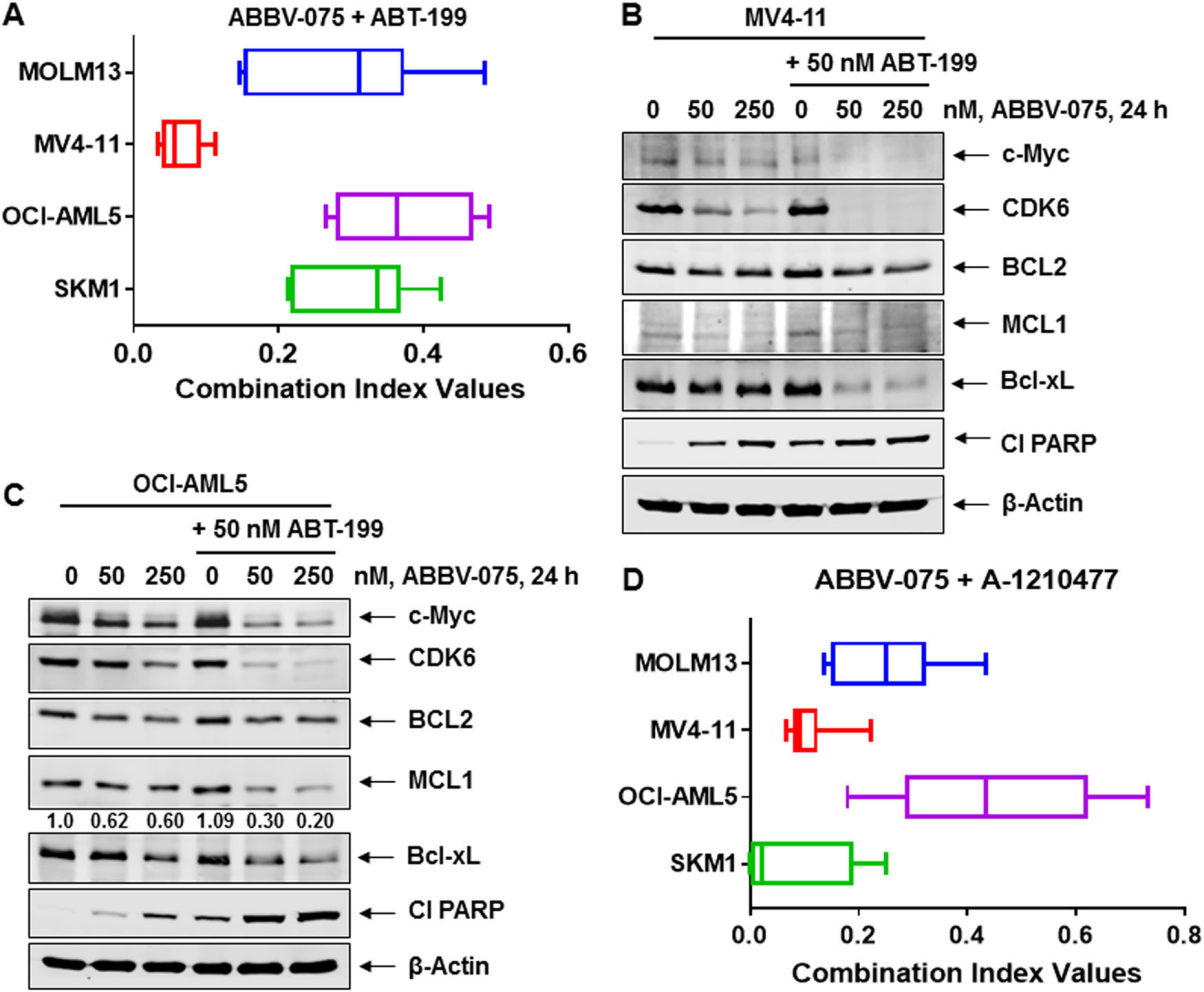
Cotreatment with ABBV-075 and ABT-199 or MCL1 inhibitor A-1210477 exerts synergistic lethal activity against AML cells. **a** MOLM13, MV4-11, OCI-AML5, and SKM1 cells were treated with ABBV-075 (dose range: 20–250 nM) and ABT-199 (dose range: 10–100 nM) for 48 h. Following this, the % of annexin V-positive, apoptotic cells was determined by flow cytometry. Combination index values were calculated by Compusyn and graphed utilizing GraphPad V7. **b**, **c** MV4-11 and OCI-AML5 cells were treated with the indicated concentrations of ABBV-075 and/or ABT-199 for 24 h. Total cell lysates were prepared and immunoblot analyses were conducted. The expression levels of β-Actin in the cell lysates served as the loading control. **d** MOLM13, MV4-11, OCI-AML5, and SKM1 cells were treated with ABBV-075 (dose range: 20–250 nM) and A-1210477 (dose range: 1–10 µM) for 48 h. Following this, the % of annexin V-positive, apoptotic cells was determined by flow cytometry. Combination index values were determined by Compusyn and graphed utilizing GraphPad V7

We first determined the in vitro lethal activity of combined exposure to various concentrations of ABBV-075 and venetoclax for 48 h, as compared to each agent alone, in AML cells (Fig. S6A to S6D). Notably, cotreatment with ABBV-075 and venetoclax synergistically induced apoptosis of MOLM13, MV4-11, and OCI-AML5 cells (CI values of <1.0) (Fig. 4a). The combination was also synergistically lethal against SKM1 cells, which were resistant to apoptosis induced by venetoclax alone (Figs. 3a and 4a). ABBV-075 treatment reduced the levels of MCL1, Bcl-xL, MYC, and CDK6, while increasing HEXIM1, p21, p27, and cleaved PARP levels in SKM1 cells (Fig. S5G). The combination of ABBV-075 and venetoclax was also effective in reducing MCL1, Bcl-xL, and BCL-2 protein levels in MV4-11 and OCI-AML5 cells (Fig. 4b, c). Although protein levels of BAX and BAK were unaltered (Fig. S7A), cleaved PARP levels increased significantly in association with the synergistic induction of apoptosis (Fig. 4b, c). Cotreatment with ABBV-075 and venetoclax also markedly reduced MYC and CDK6 levels, undermining their likely progrowth and prosurvival effects in the AML cells (Fig. 4b, c). We next determined the effects of combined treatment with ABBV-075 and A-1210477 in the AML cells. As shown, coexposure to ABBV-075 and A-1210477 at various concentrations synergistically induced apoptosis not only in MOLM13 and MV4-11 but also in OCI-AML5 and SKM1 cells (CI values of <1.0) (Fig. 4d and S7B to S7E), which were resistant to apoptosis induced by A-1210477 alone (Fig. 3d). This may not only be because ABBV-075 treatment alone reduces MCL1, BCL2, MYC, and CDK6 levels, but also because it increases p21, p27, and HEXIM1 levels in AML cells (Fig. 2e, f and S2D). Additionally, cotreatment with A-1210477 and ABBV-075, vs. treatment with each drug alone, caused greater induction in p21, BIM, and cleaved PARP levels, while attenuating c-Myc levels in OCI-AML5 cells (Fig. S7F). We also determined the lethal activity of cotreatment with venetoclax and A-1210477 at various concentrations in AML cells (Fig. S8A). As shown, cotreatment with venetoclax and A-1210477 synergistically induced apoptosis of MOLM13, MV4-11, SKM1, and OCI-AML5 cells (CI values of <1.0) (Fig. S8B).

### Cotreatment with ABBV-075 and venetoclax or A-1210477 synergistically induces in vitro lethality in PD CD34+ AML BPCs

**Fig. 5:**
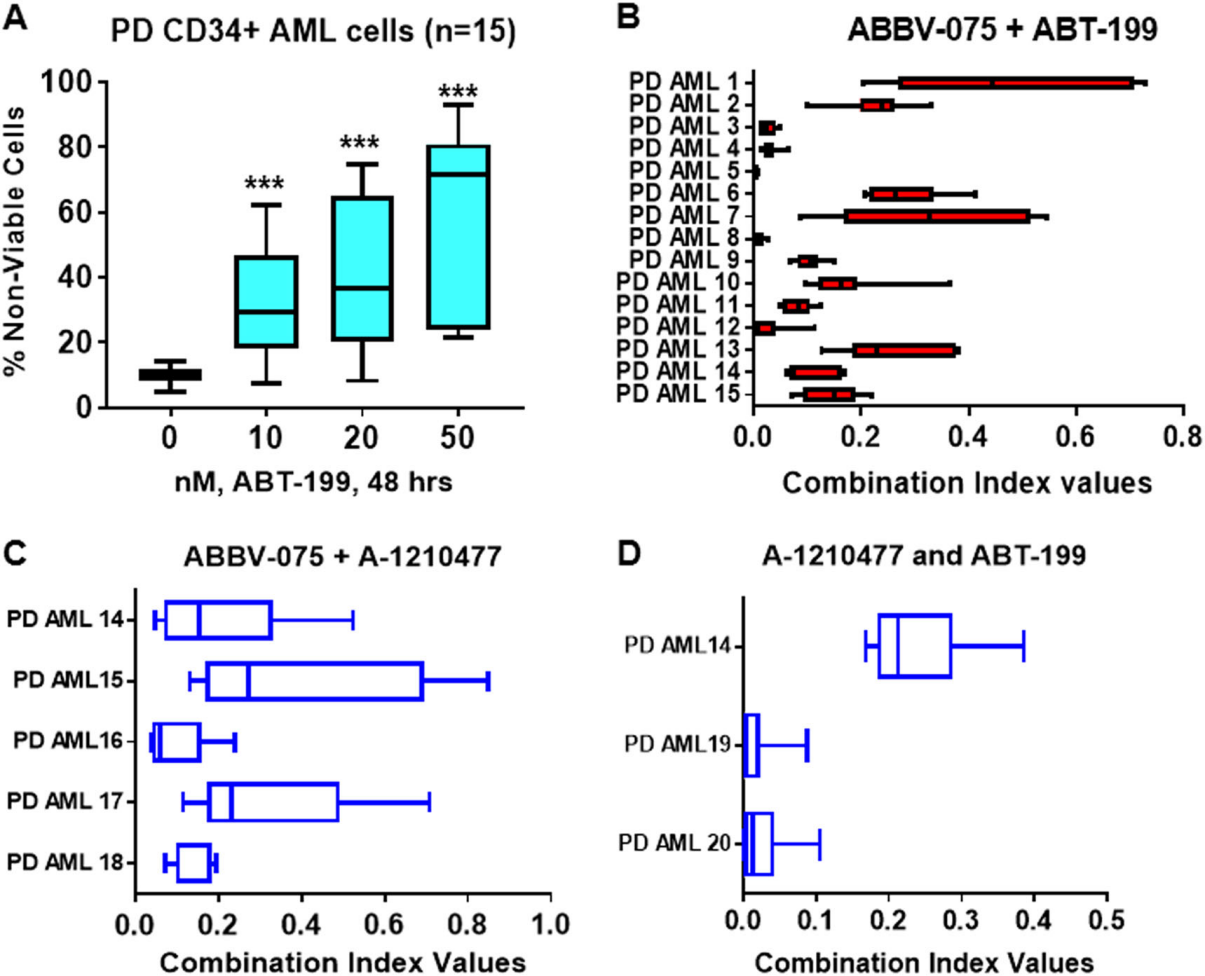
Cotreatment with ABBV-075 and ABT-199 or MCL1 inhibitor exerts synergistic lethal activity against patient-derived (PD) AML BPCs. **a** PD, CD34+ AML BPCs were treated with the indicated concentrations of ABT-199 for 48 h. At the end of treatment, cells were stained with To-Pro-3 iodide and the % of nonviable cells were determined by flow cytometry. A box plot was generated utilizing GraphPad V7. *** = *p* < 0.005 compared to the untreated control cells. **b** PD, CD34+ AML BPCs (*n* = 15) were treated with ABBV-075 (dose range: 20–250 nM) and ABT-199 (dose range: 10–100 nM) for 48 h. Following this, the % of nonviable cells was determined by flow cytometry. Combination index values were calculated by Compusyn and graphed utilizing GraphPad V7. **c** PD, CD34+ AML BPCs (*n* = 5) were treated with ABBV-075 (dose range: 20–250 nM) and A-1210477 (dose range: 1–10 µM) for 48 h. Following this, the % of nonviable cells was determined by flow cytometry. Combination index values were calculated by Compusyn and graphed utilizing GraphPad V7. **d** PD CD34+ AML BPCs (*n* = 3) were treated with A-1210477 (dose range: 2–10 µM) and ABT-199 (dose range: 10–50 nM) for 48 h. Following this, the % of nonviable cells was determined by flow cytometry. Combination index values were calculated by Compusyn and graphed utilizing GraphPad V7. BPC blast progenitor cell

Utilizing the 15 samples of PD CD34+ AML BPCs, exhibiting genetic mutations shown in Fig. S4G, we first determined the lethal effects of exposure to venetoclax alone over 48 h. In a dose-dependent manner, venetoclax treatment significantly increased loss-of-viability in these cells (Fig. 5a). Notably, exposure to a range of concentrations of ABBV-075 and venetoclax synergistically induced apoptosis in the 15 samples of CD34+ AML BPCs (CI values < 1.0) (Fig. 5b and S9). We also determined the lethal activity of cotreatment with a range of concentrations of ABBV-075 and A-1210477 in five of the samples (#14−18) of PD CD34+ AML BPCs (Figs. S4G and S10). As shown in Fig. 5c, cotreatment with ABBV-075 and A-1210477 synergistically induced loss-of-viability in the AML BPCs (CI values < 1.0). It is also noteworthy that the combination of ABT-199 and A-1210477, involving exposure to concentrations of each drug documented in Fig. S11 also synergistically induced loss-of-viability in three samples of PD CD34+ AML BPCs (Fig. 5d and S4G). We also determined the lethal activity of cotreatment with ABBV-075 and ABT-199 or A-1210477 against normal CD34+ HPCs. As shown in Fig. S12A and S12B, cotreatment with ABBV-075 and ABT-199 or A-1210477 did not induce significant loss-of-viability in normal CD34+ HPCs.

### Superior in vivo activity of cotreatment with ABBV-075 and venetoclax vs. each agent alone against AML cells

**Fig. 6:**
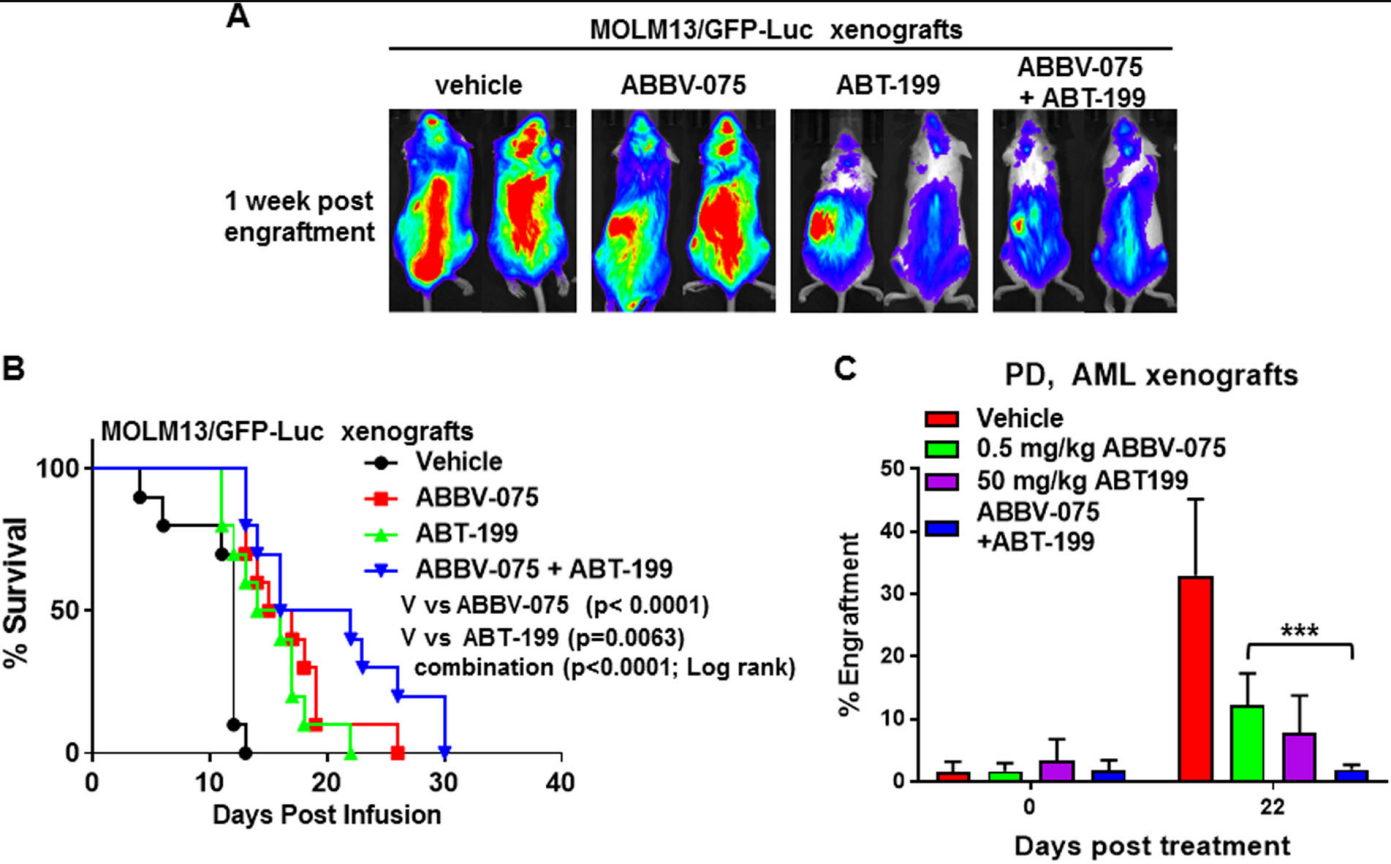
Cotreatment with ABBV-075 and ABT-199 improves survival of NSG mice-bearing AML xenografts. **a** MOLM13/GFP-Luc cells (2×10^6^) were introduced into the tail vein of pre-irradiated (2.5 Gy) NSG mice (*n* = 7 per cohort). Mice were monitored for 4 days and then treated with ABBV-075 and/or ABT-199 for 2 weeks. Mice were imaged after 1 week of treatment utilizing a xenogen camera. **b** Kaplan−Meier survival curve of NSG mice-bearing MOLM13/GFP-Luc xenografts treated with ABBV-075 and/or ABT-199. **c** Patient-derived AML xenografts (PDX) were implanted into NSG mice (*n* = 8 mice per cohort). Following documentation of engraftment, mice were treated with the indicated doses of ABBV-075 and/or ABT-199 for 22 days. Following this, the % of AML engraftment in the mice was determined by flow cytometry. *** = *p* < 0.005 compared to ABBV-075-treated mice. NSG NOD-scid IL2Rgamma^null^

We next determined the in vivo anti-AML activity of ABBV-075 and/or venetoclax against the MOLM13/GFP-Luc cell xenograft in NSG mice. Five days after tail vein infusion and engraftment of MOLM13/GFP-Luc cells, treatment with vehicle alone or ABBV-075 and/or venetoclax was started. As shown in Fig. 6a, as compared to treatment with vehicle control or ABBV-075 alone, treatment with venetoclax alone or cotreatment with ABBV-075 and venetoclax was more effective in reducing the AML xenograft growth 1-week post-engraftment of MOLM13/GFP-Luc cells (Fig. 6a, b). Combined treatment with ABBV-075 and venetoclax, vs. each agent alone also significantly improved the median and overall survival of NSG mice engrafted with MOLM13/GFP-Luc cells (*p* < 0.001), as shown in the Kaplan-Meier plot depicting the survival of the mice (Fig. 6b). Notably, as compared to vehicle control or ABBV-075 alone, cotreatment with ABBV-075 and venetoclax was also significantly more effective in reducing growth of a PDX model of AML cells (45,XX,t(3;12)(q26.1;p13),-7 with NRAS mutation) engrafted in NSG mice (Fig. 6c).

## Discussion

**Fig. 7:**
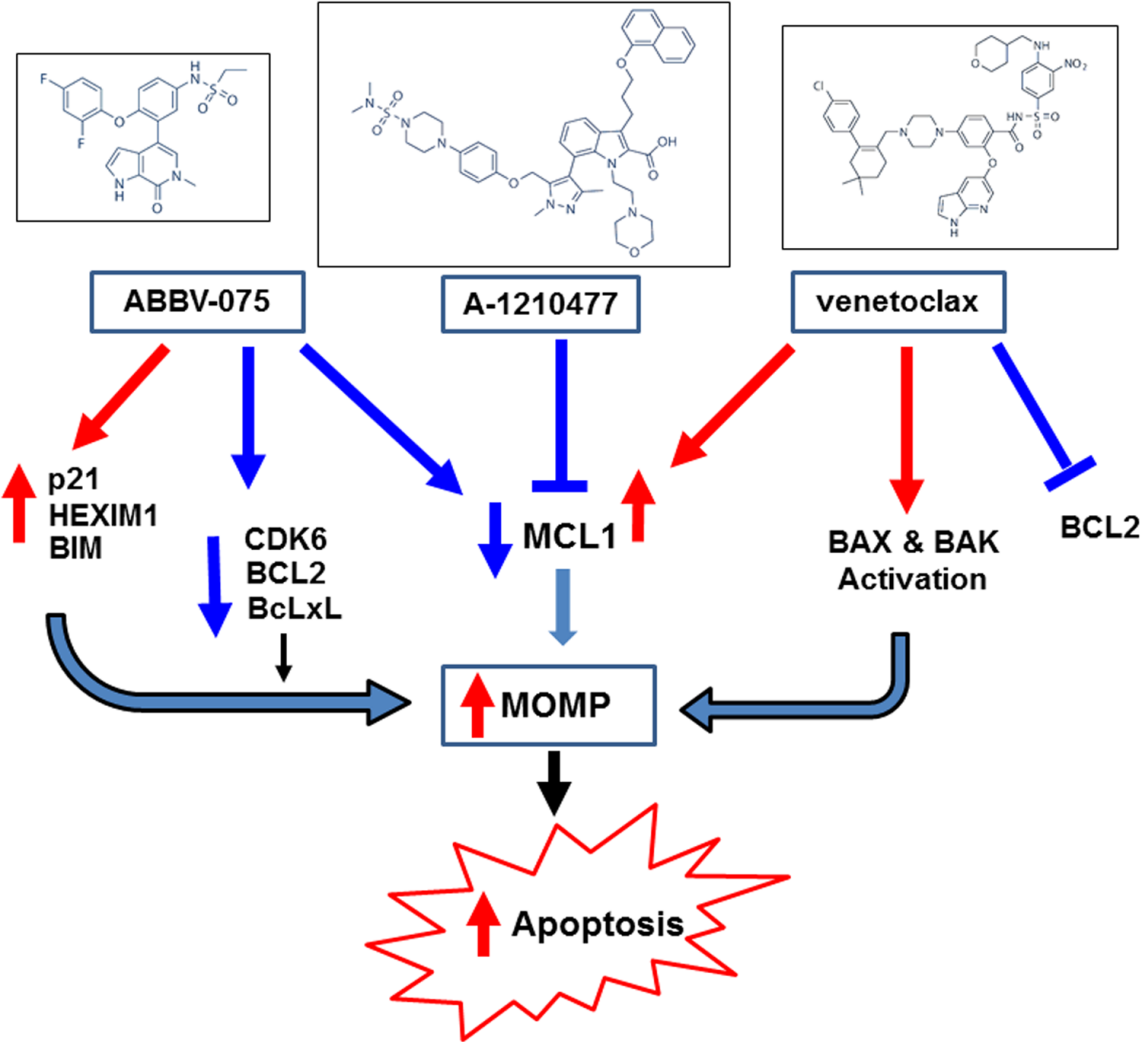
Proposed model of the antileukemia activity of ABBV-075 and ABT-199 or A-1210477 against AML cells. ABBV-075 depletes the expression of MCL1 and super-enhancer-driven oncogenes CDK6, BCL2 and BclxL with concomitant induction of the expression of p21, HEXIM1, and BIM in the AML cells. This leads to increased mitochondrial outer membrane permeabilization (MOMP) and induction of apoptosis in the AML cells. Treatment with A-1210477 blocks the activity of MCL1 in the AML cells which also leads to increased MOMP and induction of apoptosis. Treatment with venetoclax inhibits the activity of BCL2 and activates conformation change of BAX and BAK leading to increased MOMP and induction of apoptosis. Cotreatment with ABBV-075 and ABT-199 or A-1210477 or ABT-199 and A-1210477 exerts synergistic lethal activity resulting in greater apoptosis in AML cells than treatment with the single agents

It is well-documented that BH3 domain-only proteins, e.g., BIM and NOXA, either directly induce and activate BAX/BAK oligomers or release them from antiapoptotic BCL2, MCL1, and Bcl-xL to trigger mitochondrial outer membrane permeabilization (MOMP) and the ensuing execution phase of apoptosis in AML BPCs^22–24^. Therefore, prosurvival BCL2, MCL1, and Bcl-xL bind and sequester BAX/BAK and BIM/NOXA to inhibit the commitment and execution of apoptosis in a BAX/BAK-dependent manner in AML BPC^22–25,36^. Mitochondrial priming represents how close a leukemia cell is to apoptosis threshold and how addicted it is to specific antiapoptotic BCL-2 protein family members for survival^36–38^. Although not prospectively validated in the clinical setting, BH3-profiling has revealed mitochondrial priming and cellular addiction to prosurvival BCL2 or MCL1 in AML cells^26,36,38,39^. In contrast, antiapoptotic potency of BCL2 family of proteins was shown to primarily depend upon their stability and not on binding selectivity with the proapoptotic members^40^. Recently, measurements of cellular tolerance to levels of BH3 domain-only proteins, based on relative contributions of anti- and proapoptotic protein family members, was shown to predict susceptibility to apoptosis induced by antileukemia drugs and combinations^41^. Consistent with above, previous reports have highlighted that increase in levels and addiction to MCL1 and/or Bcl-xL confers innate or acquired resistance to venetoclax in leukemia and lymphoma cells (Fig. 7)^29,30^. This could be overcome by simultaneously targeting MCL1 and/or Bcl-xL (Fig. 7)^29–31,42,43^. Here, in AML BPCs, we demonstrate for the first time that treatment with the BETi ABBV-075 reduces MCL1 and Bcl-xL levels, while inducing BIM levels, thereby increasing activated BAX and/or BAK levels and undermining the role of MCL1 and Bcl-xL in mediating resistance to venetoclax-induced apoptosis (Fig. 7). This is further supported by our findings that whereas SKM1 cells were resistant to apoptosis induced by venetoclax alone, they were sensitive to ABBV-075 alone, and cotreatment with ABBV-075 and venetoclax synergistically induced apoptosis of SKM1 cells. Additionally, since similar to other BETis, ABBV-075 treatment attenuated MYC and CDK6, while simultaneously inducing HEXIM1 and CDKN1A (p21)^11,12,44–47^; these perturbations also likely augmented venetoclax-mediated inhibition of growth and survival of AML BPCs. Notably, the synergistic lethal activity of cotreatment with ABBV-075 and venetoclax was observed not only against cultured cell lines but also against PD CD34+ BPCs exhibiting diverse genetic alterations. It is also noteworthy that the concentrations of ABBV-075 and venetoclax utilized in present studies demonstrating synergy of the combination are achievable in vivo and clinically and therefore relevant^46,48^. Additionally, the dose of each drug used alone and in combination in the vivo studies was safe and exhibited superior efficacy. Recently, a mutation in the BH3-peptide-binding hydrophobic pocket of BCL2 that reduces venetoclax binding has been shown to confer resistance to venetoclax in cultured cell lines^29,49^. However, the clinical relevance of this mechanism of resistance is uncertain, since it has not been documented in PD AML BPCs. Taken together, our findings support further clinical evaluation of combination therapy of AML with ABBV-075 and venetoclax. Previous clinical studies of BCL2/Bcl-xL inhibitor navitoclax in patients with CLL and small cell lung cancer demonstrated thrombocytopenia as a major dose-limiting toxicity, which would militate against the use of navitoclax in combination with ABBV-075^50,51^.

Consistent with previous reports, our findings also show that A-1210477 treatment increased MCL1 levels^34,35^. Stability of MCL1 is controlled by ubiquitylation by the E3 ligase MULE (MCL1 ubiquitin ligase E3) and deubiquitinase USP9X^52,53^. By displacing BH3 domain-containing MULE from MCL1, A-1210477 is known to stabilize and increase MCL1 levels^23,34,35^. However, as shown here and previously in other reports, treatment with BETi, including ABBV-075, inhibits RNAP2-mediated transcription and levels of MCL1 and Bcl-xL, while simultaneously inducing BIM levels^12,17,44,46^. Together these perturbations due to superior lethal activity of cotargeting MCL1 and BCL2 is also underscored here by our findings that cotreatment with A-1210477 and venetoclax induces synergistic lethal effects against AML BPCs. Similar to the effects of ABBV-075, previous reports have also documented that cotreatment with CDK9 inhibitor that transcriptionally reduces short-lived MCL1 levels also enhances lethal effects of BCL2 inhibition^54^.

Several recent reports have also presented evidence for superior activity of combining venetoclax with other conventional or novel anti-AML agents, besides the BETi described here, that further reduce the threshold for apoptosis in AML BPCs. These include combinations with standard anti-AML agents, e.g., azacytidine^32^, cytarbine and idarubicin^30^, which induce mitochondrial priming by inducing the expressions of the BH3 domain-only proapoptosis proteins such as BIM, NOXA, and PUMA in a p53-dependent or independent manner^41,55,56^. Combinations of venetoclax with novel anti-AML agents include inhibitors targeting MEK, PI3K/mTOR, NEDD8, HDM2, and IDH1/2^57–59^. There is evidence to suggest that venetoclax may selectively target AML stem cells that depend on mitochondrial oxidative phosphorylation^60^. If so, how combining each of these novel agents modulates this important activity of venetoclax would have to be evaluated in future studies.

## Supplementary information

Supplemental Data Figures

Supplemental Figure Legends

Extended and Supplemental Methods

## Data Availability

RNA-Seq datasets are deposited in GEO under accession number (GSE93578). Methods for sequencing of primary AML samples, reverse phase protein array, ATAC-Seq analysis and assessment of BAX and BAK conformation change are provided in the Supplemental Methods.
