# Supplementary material for: Superior efficacy of cotreatment with BET protein inhibitor and BCL2 or MCL1 inhibitor against AML blast progenitor cells
Source: Blood Cancer J. 2019 Jan 15;9(2):4. doi: 10.1038/s41408-018-0165-5 (PMC6333829; doi:10.1038/s41408-018-0165-5)

Figure S1

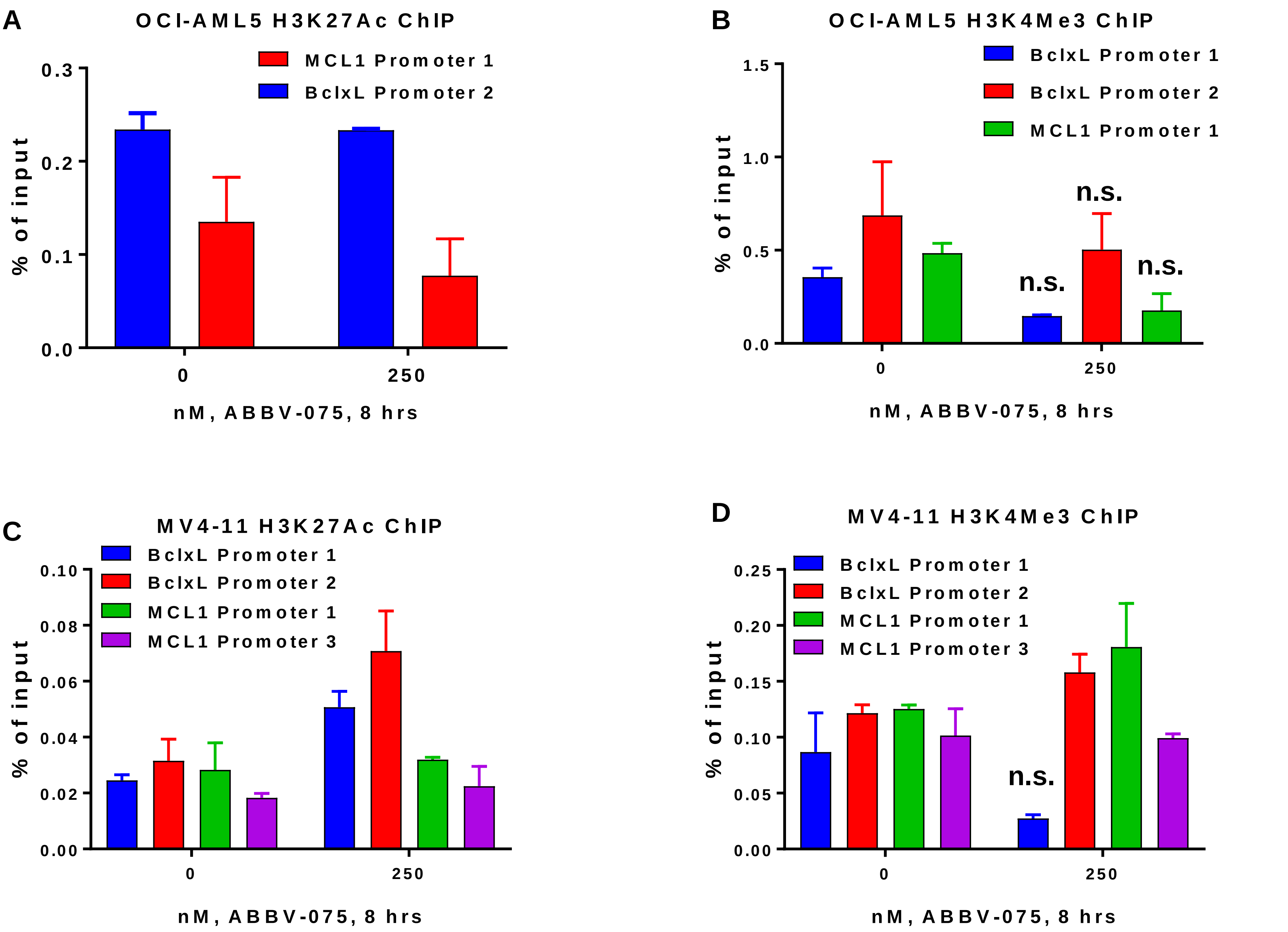
A
OCI-AML5 H3K27Ac ChIP
MCL1 Promoter 1
BclxL Promoter 2
% of input
0.0
0.1
0.2
0.3
0
250
nM, ABBV-075, 8 hrs
B
OCI-AML5 H3K4Me3 ChIP
BclxL Promoter 1
BclxL Promoter 2
MCL1 Promoter 1
% of input
0.0
0.5
1.0
1.5
n.s.
n.s.
n.s.
0
250
nM, ABBV-075, 8 hrs
C
MV4-11 H3K27Ac ChIP
BclxL Promoter 1
BclxL Promoter 2
MCL1 Promoter 1
MCL1 Promoter 3
% of input
0.00
0.02
0.04
0.06
0.08
0.10
0
250
nM, ABBV-075, 8 hrs
D
MV4-11 H3K4Me3 ChIP
BclxL Promoter 1
BclxL Promoter 2
MCL1 Promoter 1
MCL1 Promoter 3
% of input
0.00
0.05
0.10
0.15
0.20
0.25
n.s.
0
250
nM, ABBV-075, 8 hrs

Figure S2

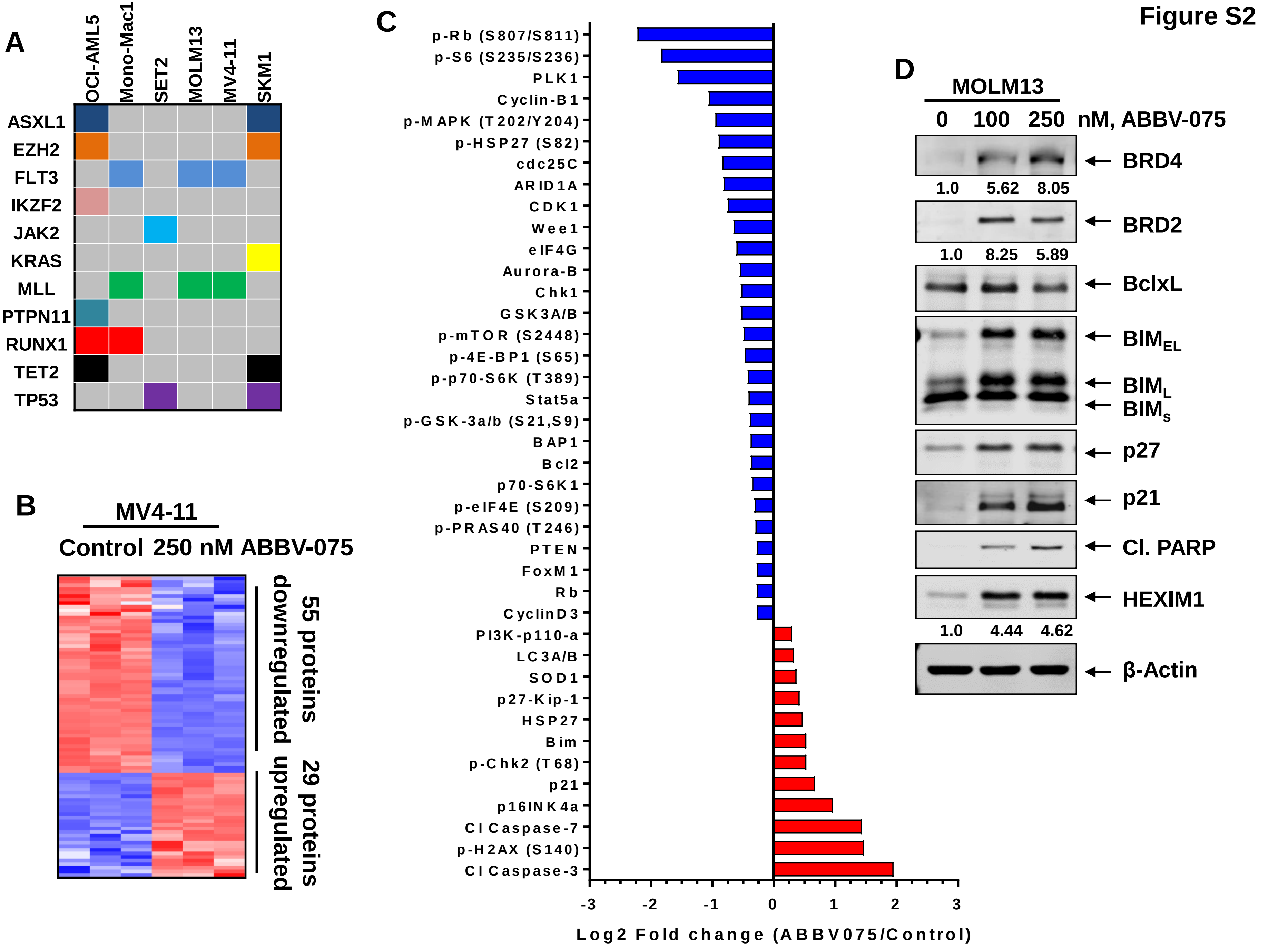
A
OCI-AML5
Mono-Mac1
SET2
MOLM13
MV4-11
SKM1
ASXL1
EZH2
FLT3
IKZF2
JAK2
KRAS
MLL
PTPN11
RUNX1
TET2
TP53
B
MV4-11
Control
250 nM ABBV-075
55 proteins downregulated
29 proteins upregulated
C
p-Rb (S807/S811)
p-S6 (S235/S236)
PLK1
Cyclin-B1
p-MAPK (T202/Y204)
p-HSP27 (S82)
cdc25C
ARID1A
CDK1
Wee1
eIF4G
Aurora-B
Chk1
GSK3A/B
p-mTOR (S2448)
p-4E-BP1 (S65)
p-p70-S6K (T389)
Stat5a
p-GSK-3a/b (S21,S9)
BAP1
Bcl2
p70-S6K1
p-eIF4E (S209)
p-PRAS40 (T246)
PTEN
FoxM1
Rb
CyclinD3
PI3K-p110-a
LC3A/B
SOD1
p27-Kip-1
HSP27
Bim
p-Chk2 (T68)
p21
p16INK4a
Cl Caspase-7
p-H2AX (S140)
Cl Caspase-3
-3
-2
-1
0
1
2
3
Log2 Fold change (ABBV075/Control)
D
MOLM13
0
100
250
nM, ABBV-075
BRD4
1.0
5.62
8.05
BRD2
1.0
8.25
5.89
BclxL
BIM$_{EL}$
BIM$_{L}$
BIM$_{S}$
p27
p21
Cl. PARP
HEXIM1
1.0
4.44
4.62
β-Actin

Figure S3

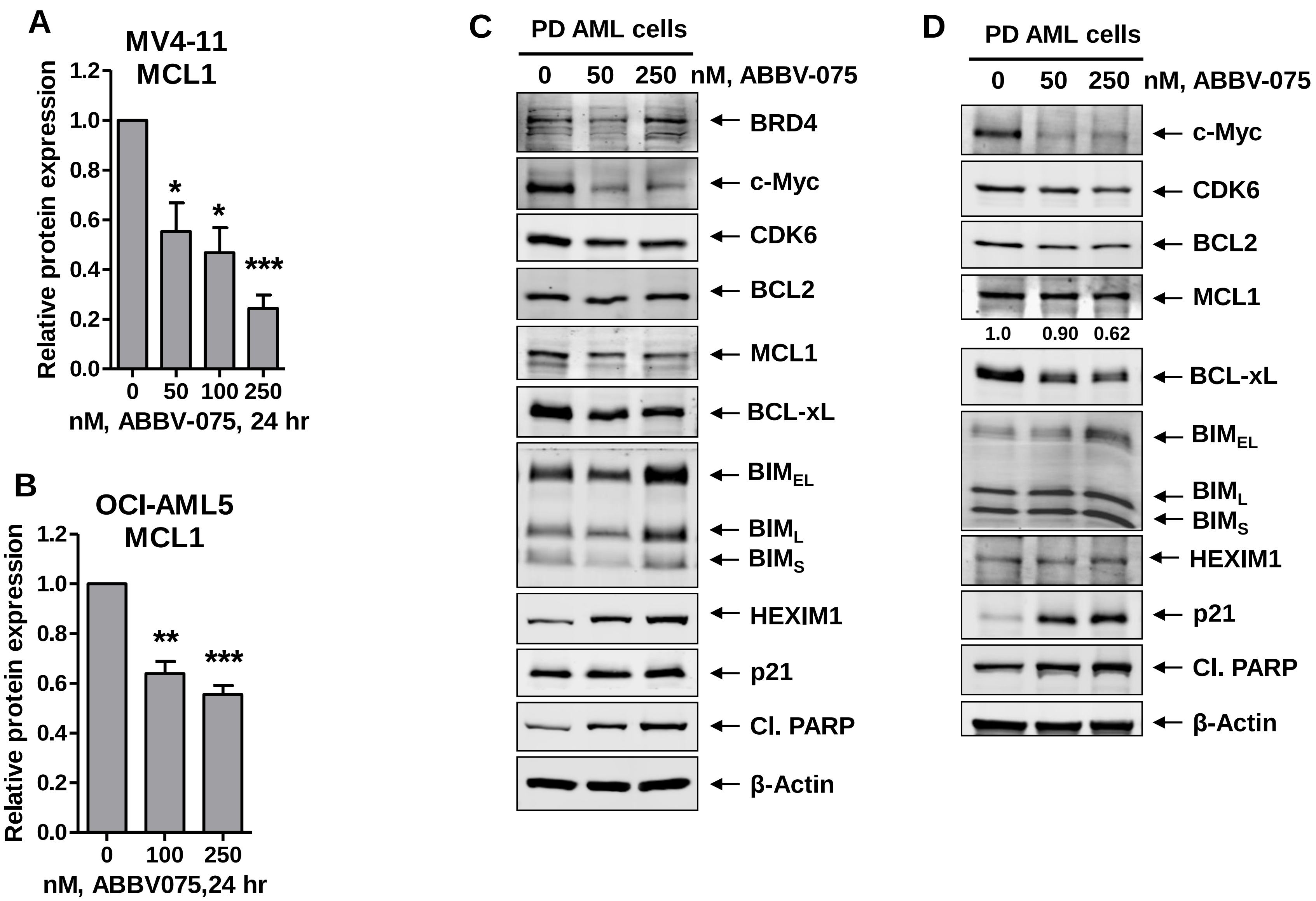
A
MV4-11
MCL1
Relative protein expression
1.2
1.0
0.8
0.6
0.4
0.2
0.0
*
*
***
0
50
100
250
nM, ABBV-075, 24 hr
B
OCI-AML5
MCL1
Relative protein expression
1.2
1.0
0.8
0.6
0.4
0.2
0.0
**
***
0
100
250
nM, ABBV075,24 hr
C
PD AML cells
0 50 250 nM, ABBV-075
BRD4
c-Myc
CDK6
BCL2
MCL1
BCL-xL
BIMEL
BIML
BIMS
HEXIM1
p21
Cl. PARP
β-Actin
D
PD AML cells
0 50 250 nM, ABBV-075
c-Myc
CDK6
BCL2
MCL1
1.0 0.90 0.62
BCL-xL
BIMEL
BIML
BIMS
HEXIM1
p21
Cl. PARP
β-Actin

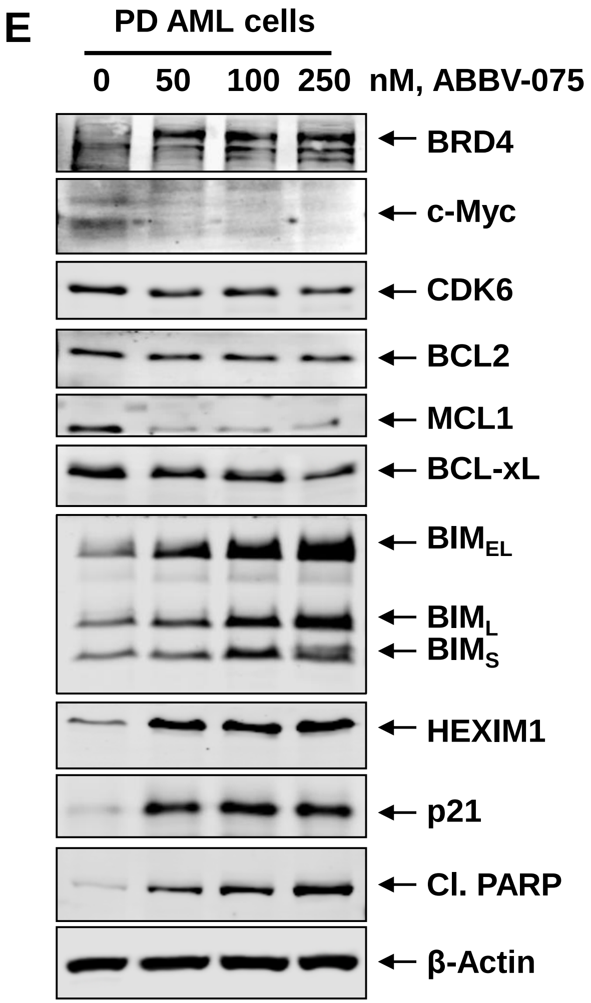
E
PD AML cells
0
50
100
250
nM, ABBV-075
BRD4
c-Myc
CDK6
BCL2
MCL1
BCL-xL
BIM EL
BIM L
BIM S
HEXIM1
p21
Cl. PARP
β-Actin

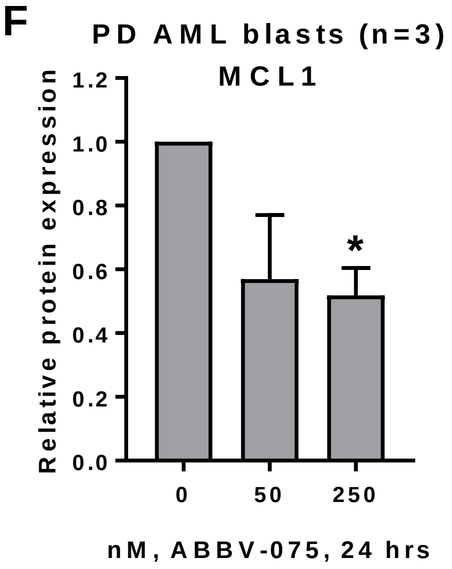
F
PD AML blasts (n=3)
MCL1
Relative protein expression
1.2
1.0
0.8
0.6
0.4
0.2
0.0
*
0
50
250
nM, ABBV-075, 24 hrs

Figure S3

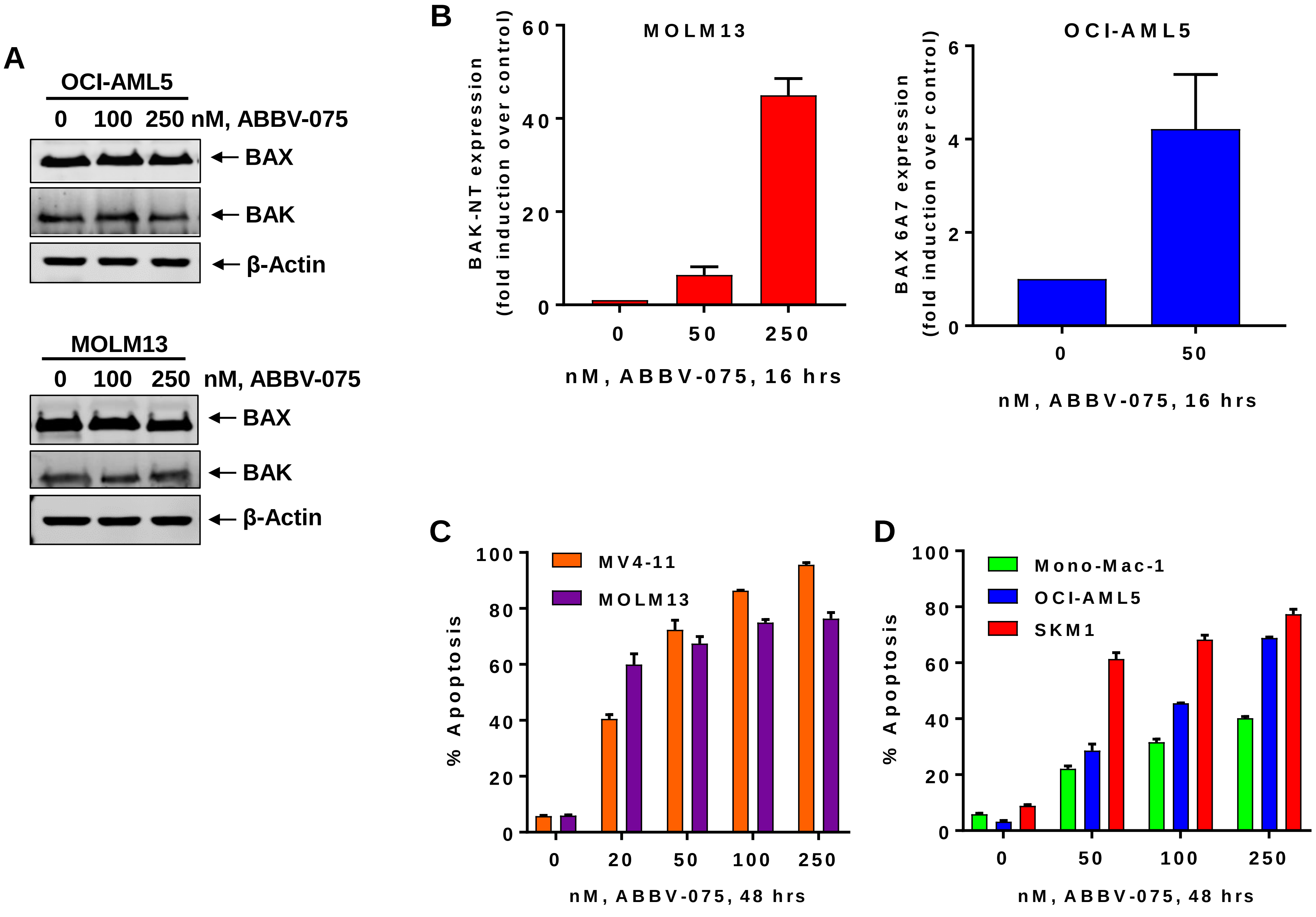
A
OCI-AML5
0 100 250 nM, ABBV-075
BAX
BAK
β-Actin
MOLM13
0 100 250 nM, ABBV-075
BAX
BAK
β-Actin
B
MOLM13
BAK-NT expression (fold induction over control)
nM, ABBV-075, 16 hrs
OCI-AML5
BAX 6A7 expression (fold induction over control)
nM, ABBV-075, 16 hrs
C
MV4-11
MOLM13
% Apoptosis
nM, ABBV-075, 48 hrs
D
Mono-Mac-1
OCI-AML5
SKM1
% Apoptosis
nM, ABBV-075, 48 hrs

Figure S4

Figure S4

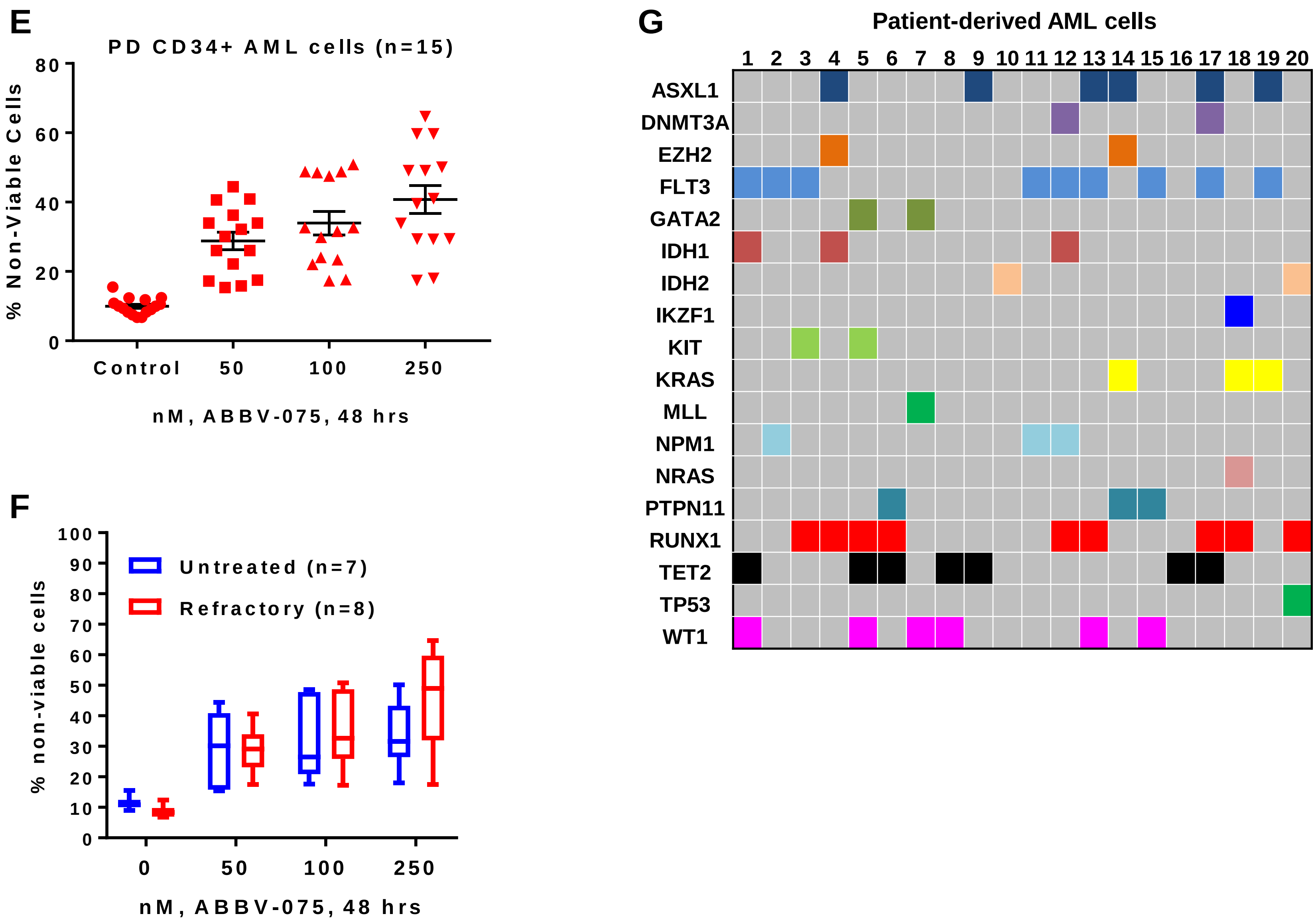

E
PD CD34+ AML cells (n=15)
% Non-Viable Cells
0
20
40
60
80
Control
50
100
250
nM, ABBV-075, 48 hrs
F
% non-viable cells
0
10
20
30
40
50
60
70
80
90
100
Untreated (n=7)
Refractory (n=8)
0
50
100
250
nM, ABBV-075, 48 hrs
G
Patient-derived AML cells
1 2 3 4 5 6 7 8 9 10 11 12 13 14 15 16 17 18 19 20
ASXL1
DNMT3A
EZH2
FLT3
GATA2
IDH1
IDH2
IKZF1
KIT
KRAS
MLL
NPM1
NRAS
PTPN11
RUNX1
TET2
TP53
WT1

Figure S5

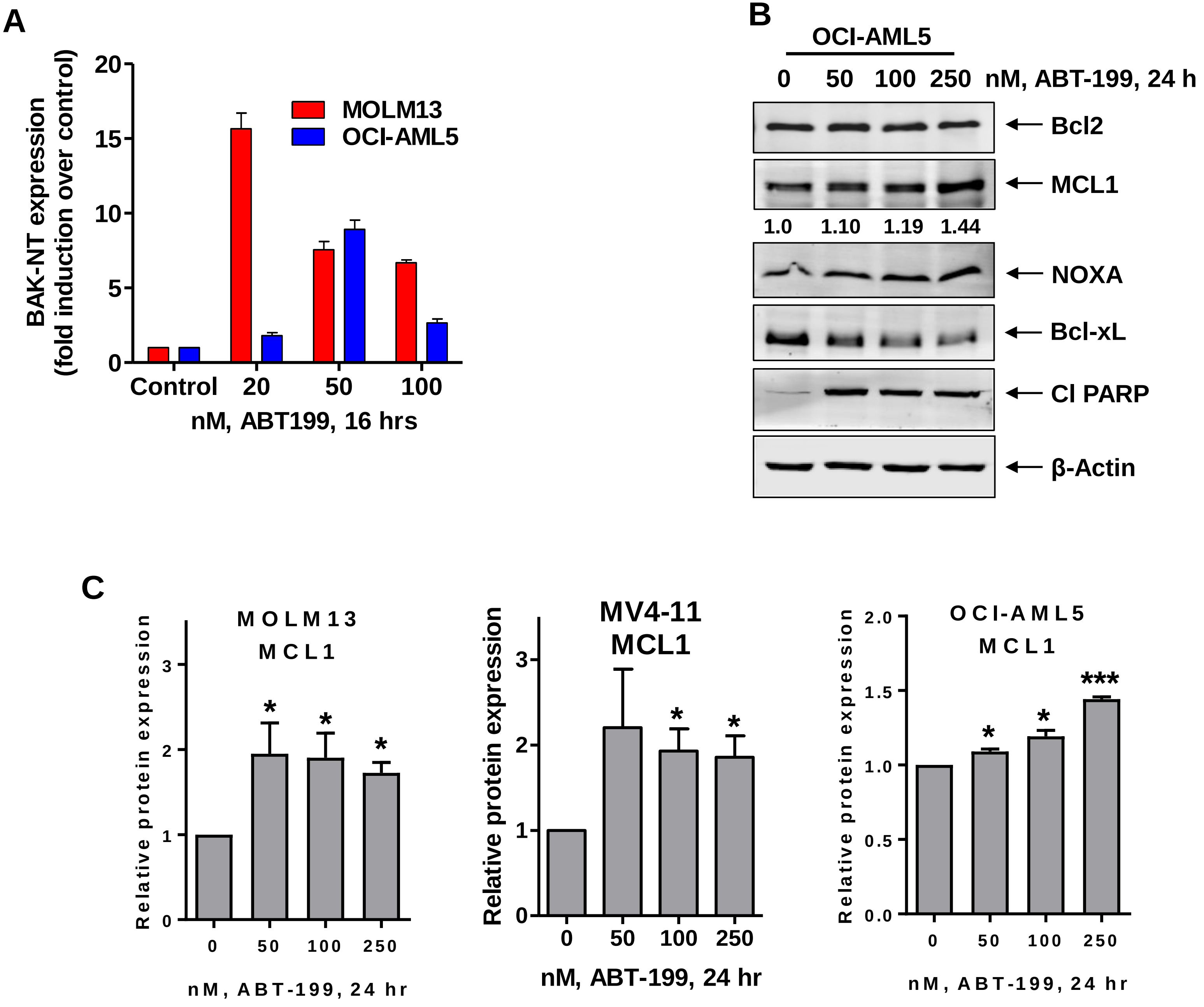
A
BAK-NT expression (fold induction over control)
20
15
10
5
0
MOLM13
OCI-AML5
Control
20
50
100
nM, ABT199, 16 hrs
B
OCI-AML5
0 50 100 250 nM, ABT-199, 24 h
Bcl2
MCL1
1.0 1.10 1.19 1.44
NOXA
Bcl-xL
Cl PARP
β-Actin
C
MOLM13
MCL1
Relative protein expression
0 50 100 250
nM, ABT-199, 24 hr
MV4-11
MCL1
Relative protein expression
0 50 100 250
nM, ABT-199, 24 hr
OCI-AML5
MCL1
Relative protein expression
2.0
1.5
1.0
0.5
0.0
0 50 100 250
nM, ABT-199, 24 hr

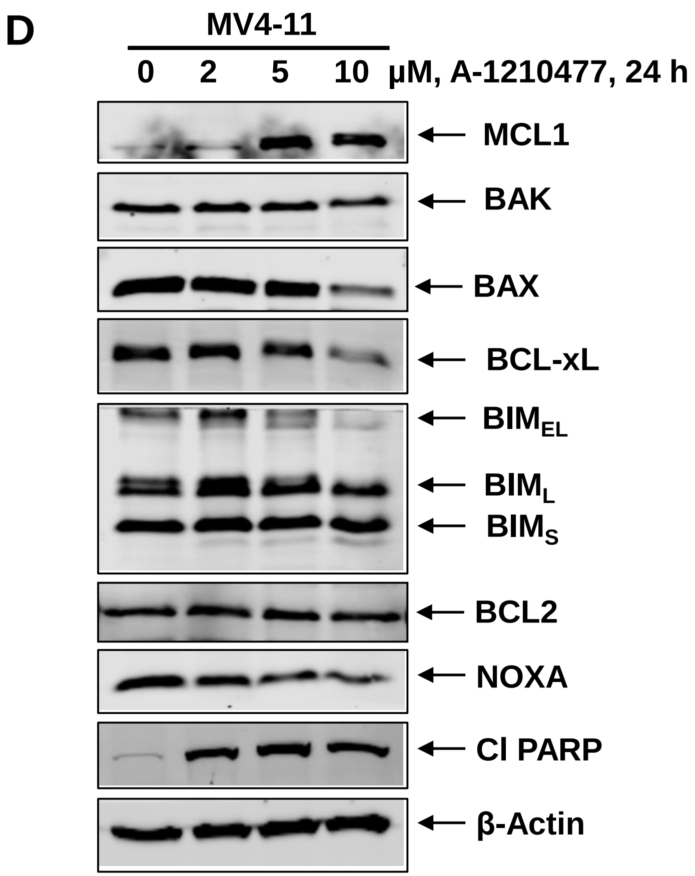
D
MV4-11
0 2 5 10 μM, A-1210477, 24 h
MCL1
BAK
BAX
BCL-xL
BIM$_{EL}$
BIM$_L$
BIM$_S$
BCL2
NOXA
Cl PARP
β-Actin

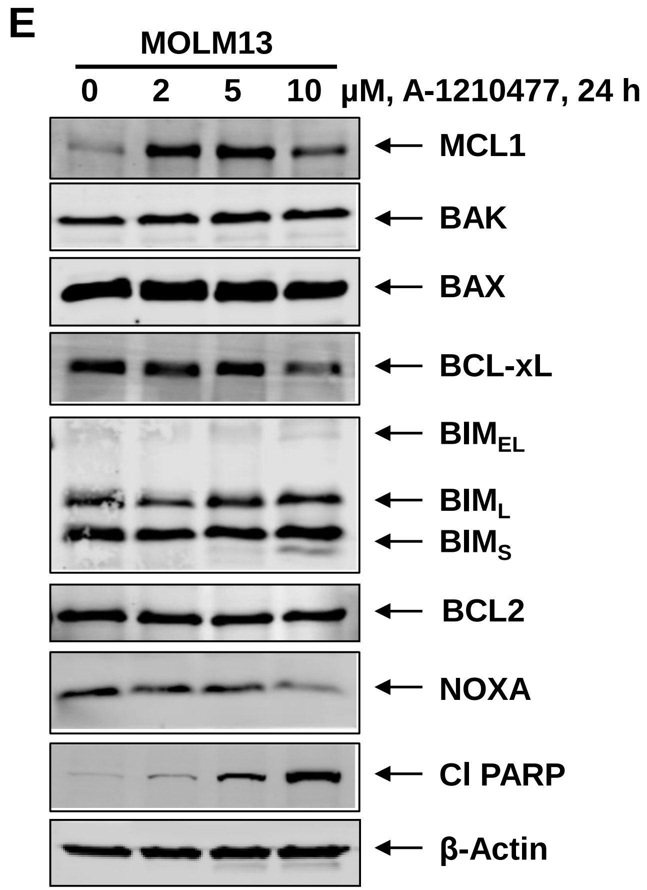
E
MOLM13
0 2 5 10 μM, A-1210477, 24 h
MCL1
BAK
BAX
BCL-xL
BIM$_{EL}$
BIM$_L$
BIM$_S$
BCL2
NOXA
Cl PARP
β-Actin

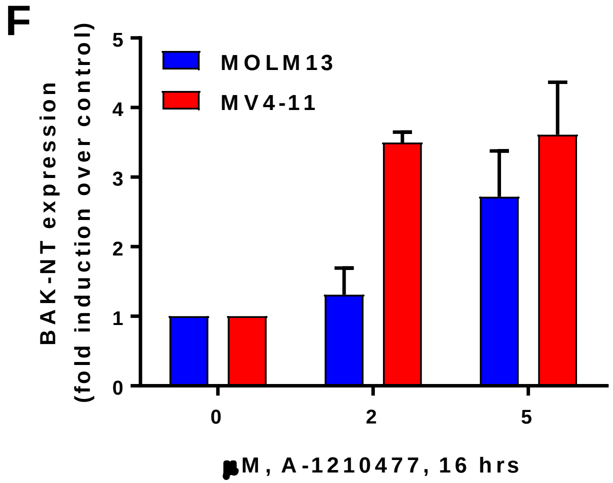
F
MOLM13
MV4-11
BAK-NT expression
(fold induction over control)
0
1
2
3
4
5
0
2
5
μM, A-1210477, 16 hrs

Figure S5

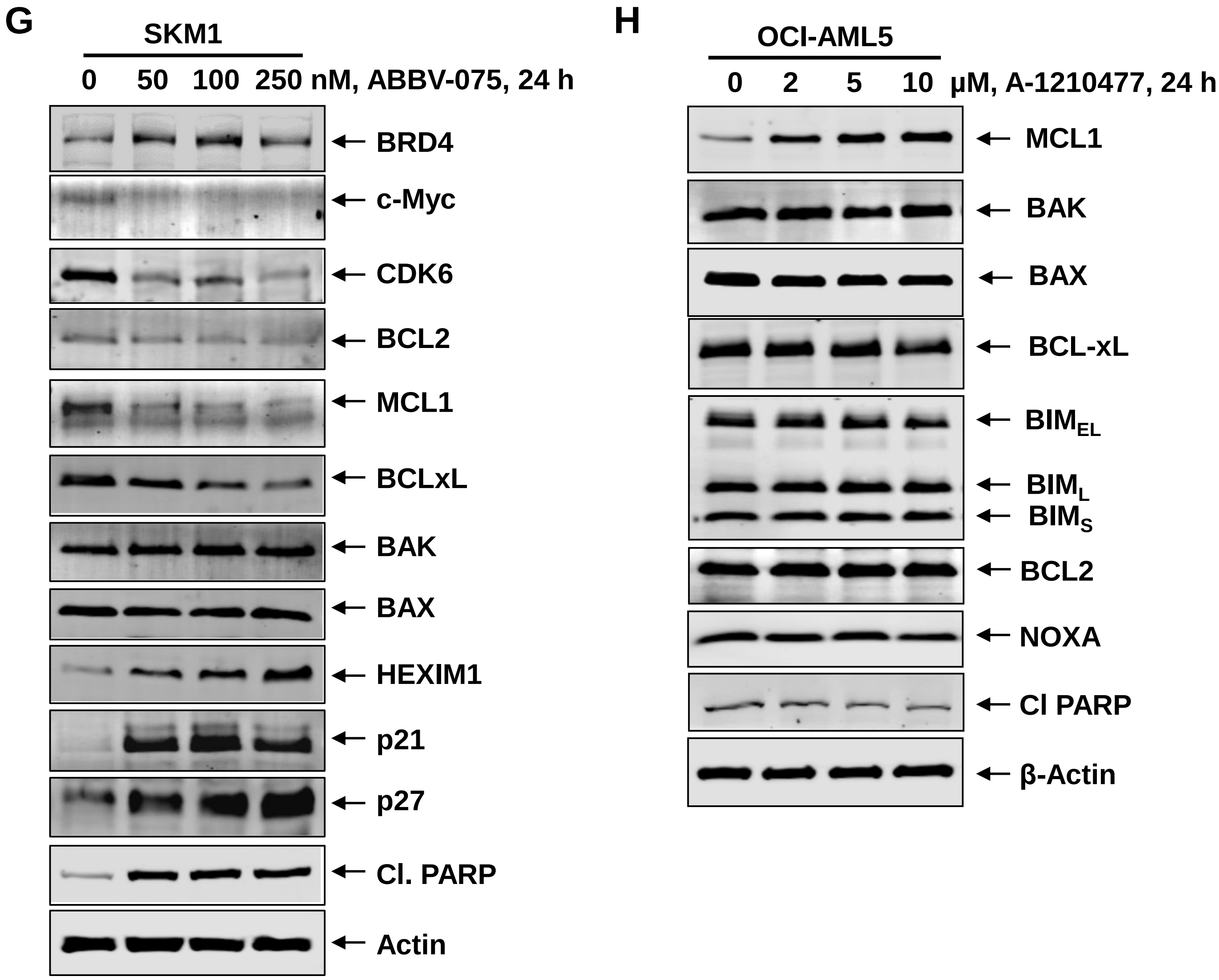
G
SKM1
0 50 100 250 nM, ABBV-075, 24 h
BRD4
c-Myc
CDK6
BCL2
MCL1
BCLxL
BAK
BAX
HEXIM1
p21
p27
Cl. PARP
Actin
H
OCI-AML5
0 2 5 10 μM, A-1210477, 24 h
MCL1
BAK
BAX
BCL-xL
BIM_{EL}
BIM_L
BIM_S
BCL2
NOXA
Cl PARP
β-Actin

Figure S5

Figure S6

A

MV4-11

ABBV-075 (nM)	ABT-199 (nM)	Effect	CI
10.0	20.0	0.594	0.39211
10.0	50.0	0.715	0.30182
10.0	100.0	0.8455	0.20539
20.0	20.0	0.8475	0.40478
20.0	50.0	0.889	0.33971
20.0	100.0	0.929	0.26833

B

MOLM13

ABBV-075 (nM)	ABT-199 (nM)	Effect	CI
50.0	20.0	0.534	0.33727
50.0	50.0	0.568	0.30983
50.0	100.0	0.6975	0.15305
100.0	20.0	0.5915	0.48407
100.0	50.0	0.6795	0.31280
100.0	100.0	0.8015	0.14621

C

OCI-AML5

ABBV-075 (nM)	ABT-199 (nM)	Fa	CI Value
50	50	0.691	0.27842
60	60	0.729	0.26517
70	70	0.722	0.32281
75	75	0.714	0.36310
80	80	0.702	0.41661
90	90	0.702	0.46869
100	100	0.712	0.49005

D

SKM1

ABBV-075 (nM)	ABT-199 (nM)	Fa	CI Value
20.0	100.0	0.3305	0.42311
50.0	100.0	0.548	0.32541
100.0	100.0	0.669	0.34624
20.0	200.0	0.376	0.35094
50.0	200.0	0.6305	0.21596
100.0	200.0	0.7505	0.21389

Figure S7

A

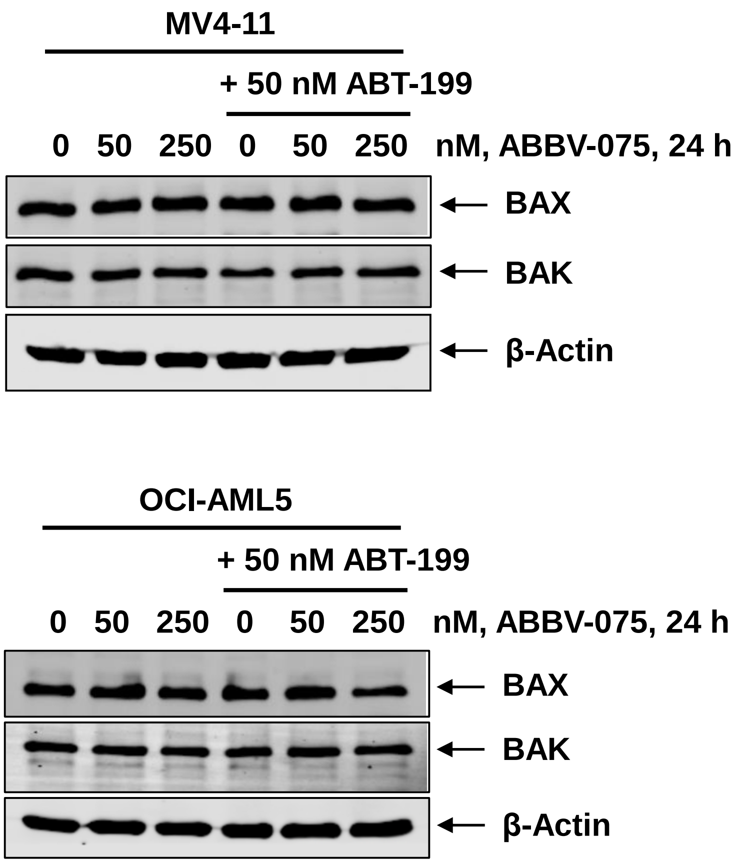

B

MOLM13

A-1210477 (μM)	ABBV-075 (nM)	Fa	CI Value
0.5	20.0	0.7088	0.27393
0.5	50.0	0.7355	0.43366
0.5	100.0	0.8841	0.14612
1.0	20.0	0.7727	0.24572
1.0	50.0	0.8214	0.25577
1.0	100.0	0.8909	0.17157
2.0	20.0	0.9186	0.13651
2.0	50.0	0.9257	0.13851
2.0	100.0	0.9278	0.15428
5.0	20.0	0.9123	0.34715
5.0	50.0	0.9193	0.33870
5.0	100.0	0.9383	0.28344

C

OCI-AML5

A-1210477 (μM)	ABBV-075 (nM)	Fa	CI Value
0.5	50.0	0.219	0.73280
0.5	100.0	0.3845	0.72301
0.5	250.0	0.7181	0.52313
1.0	50.0	0.2682	0.57865
1.0	100.0	0.4257	0.62173
1.0	250.0	0.6402	0.71792
2.0	50.0	0.3713	0.37986
2.0	100.0	0.5283	0.43200
2.0	250.0	0.7522	0.44816
5.0	50.0	0.4971	0.24116
5.0	100.0	0.6007	0.33300
5.0	250.0	0.7584	0.43510
10.0	50.0	0.5802	0.17943
10.0	100.0	0.7117	0.21513
10.0	250.0	0.8346	0.28625

D

MV4-11

A-1210477 (μM)	ABBV-075 (nM)	Fa	CI Value
1.0	10.0	0.8552	0.11331
1.0	20.0	0.9124	0.07116
1.0	50.0	0.9247	0.06649
2.0	10.0	0.8528	0.22334
2.0	20.0	0.9442	0.09085
2.0	50.0	0.9493	0.08562
5.0	10.0	0.9693	0.13312
5.0	20.0	0.9737	0.11685
5.0	50.0	0.9815	0.08697

E

SKM1

A-1210477 (μM)	ABBV-075 (nM)	Fa	CI Value
2.0	20.0	0.5	0.25028
5.0	50.0	0.5788	0.21821
2.0	100.0	0.6489	0.16344
5.0	20.0	0.6747	0.02231
5.0	50.0	0.7055	0.03459
5.0	100.0	0.7742	0.02108
10.0	20.0	0.8225	0.00155
10.0	50.0	0.8793	8.67E-4
10.0	100.0	0.9123	5.33E-4

Figure S7

Figure S7

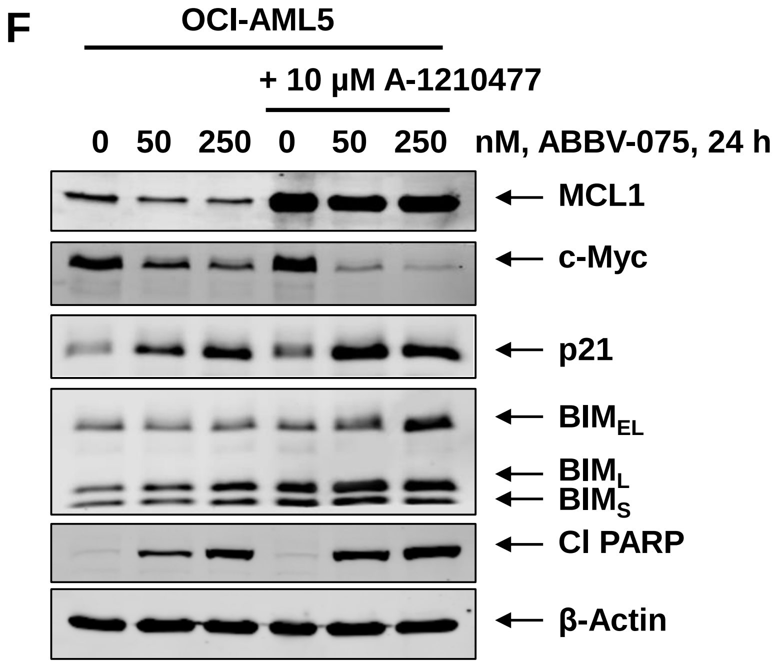
F
OCI-AML5
+ 10 μM A-1210477
0 50 250 0 50 250 nM, ABBV-075, 24 h
MCL1
c-Myc
p21
BIMEL
BIML
BIMS
Cl PARP
β-Actin

Figure S8

A

MV4-11

A-1210477 (µM)	ABT199 (nM)	Effect	CI
0.5	20.0	0.9411	0.01271
1.0	20.0	0.975	0.01108
2.0	20.0	0.9786	0.01920
0.5	50.0	0.969	0.00692
1.0	50.0	0.9785	0.00970
2.0	50.0	0.9818	0.01661

OCI-AML5

A-1210477 (µM)	ABT199 (nM)	Effect	CI
2.0	20.0	0.6965	0.19045
5.0	20.0	0.818	0.08889
10.0	20.0	0.8686	0.05740
2.0	50.0	0.7784	0.29386
5.0	50.0	0.8387	0.18839
10.0	50.0	0.9251	0.07065

MOLM13

A-1210477 (µM)	ABT199 (nM)	Effect	CI
0.5	20.0	0.5335	0.11286
1.0	20.0	0.58575	0.03314
2.0	20.0	0.66135	0.00700
0.5	50.0	0.5382	0.24749
1.0	50.0	0.58675	0.07592
2.0	50.0	0.70345	0.00485

SKM1

A-1210477 (µM)	ABT199 (nM)	Fa	CI Value
2.0	20.0	0.1432	0.60168
5.0	20.0	0.2244	0.15549
10.0	20.0	0.301	0.07683
2.0	50.0	0.1537	0.71382
5.0	50.0	0.274	0.07107
10.0	50.0	0.36	0.03297

Figure S8

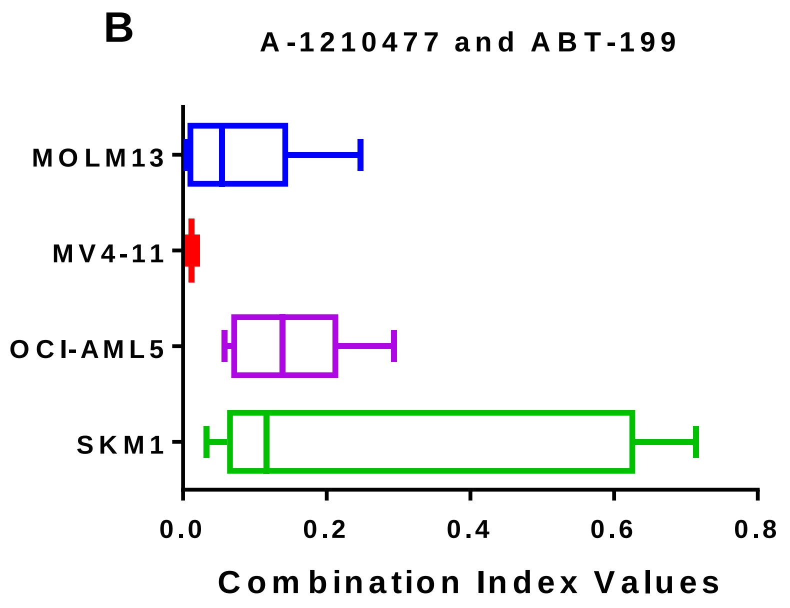
B
A-1210477 and ABT-199
MOLM13
MV4-11
OCI-AML5
SKM1
0.0
0.2
0.4
0.6
0.8
Combination Index Values

Figure S9

PD CD34+ AML

ABBV-075 (nM)	ABT-199 (nM)	Effect	CI
20.0	10.0	0.424	0.19660
50.0	10.0	0.398	0.26248
100.0	10.0	0.385	0.32964
250.0	10.0	0.501	0.09861
20.0	20.0	0.497	0.20108
50.0	20.0	0.489	0.21638
100.0	20.0	0.469	0.26051
250.0	20.0	0.471	0.25878

PD CD34+ AML

ABBV-075 (nM)	ABT-199 (nM)	Effect	CI
50.0	10.0	0.6735	0.36523
100.0	10.0	0.7287	0.20777
250.0	10.0	0.749	0.16593
50.0	20.0	0.8197	0.13496
100.0	20.0	0.7998	0.17790
250.0	20.0	0.8373	0.10356
50.0	50.0	0.8666	0.15737
100.0	50.0	0.8647	0.16297
250.0	50.0	0.891	0.09632

PD CD34+ AML

ABBV-075 (nM)	ABT-199 (nM)	Effect	CI
50.0	10.0	0.6729	0.16460
100.0	10.0	0.7745	0.14263
250.0	10.0	0.839	0.18058
50.0	20.0	0.7409	0.09617
100.0	20.0	0.8368	0.07417
250.0	20.0	0.8228	0.21799

PD CD34+ AML

ABBV-075 (nM)	ABT-199 (nM)	Effect	CI
20.0	10.0	0.6071	0.07783
50.0	10.0	0.6268	0.16982
100.0	10.0	0.7252	0.16244
20.0	20.0	0.626	0.06831
50.0	20.0	0.7598	0.06043
100.0	20.0	0.8096	0.07457

PD CD34+ AML

ABBV-075 (nM)	ABT-199 (nM)	Effect	CI
20.0	10.0	0.325	0.24874
50.0	10.0	0.3842	0.38171
100.0	10.0	0.4761	0.37597
20.0	20.0	0.3443	0.21128
50.0	20.0	0.4653	0.20398
100.0	20.0	0.6184	0.12649

Figure S9

PD CD34+ AML

ABBV-075 (nM)	ABT-199 (nM)	Effect	CI
50.0	10.0	0.2311	0.01844
100.0	10.0	0.2112	0.11381
250.0	10.0	0.2737	0.00570
50.0	20.0	0.2543	0.00724
100.0	20.0	0.2774	0.00330
250.0	20.0	0.3003	0.00182

PD CD34+ AML

ABBV-075 (nM)	ABT-199 (nM)	Effect	CI
50.0	10.0	0.9463	0.15088
100.0	10.0	0.9736	0.08590
250.0	10.0	0.9808	0.06700
50.0	20.0	0.9851	0.11007
100.0	20.0	0.9856	0.10720
250.0	20.0	0.9858	0.10605

PD CD34+ AML

ABBV-075 (nM)	ABT-199 (nM)	Effect	CI
50.0	10.0	0.544	0.09939
100.0	10.0	0.6153	0.04775
250.0	10.0	0.6056	0.05553
50.0	20.0	0.587	0.12594
100.0	20.0	0.6245	0.08519
250.0	20.0	0.6292	0.08260

PD CD34+ AML

ABBV-075 (nM)	ABT-199 (nM)	Effect	CI
50.0	10.0	0.4909	0.01570
50.0	20.0	0.539	0.00372
50.0	50.0	0.6041	7.14E-4
250.0	10.0	0.5228	0.02791
250.0	20.0	0.5639	0.00769
250.0	50.0	0.6532	4.99E-4

PD CD34+ AML

ABBV-075 (nM)	ABT-199 (nM)	Effect	CI
20.0	20.0	0.816	0.36609
50.0	20.0	0.848	0.29204
100.0	20.0	0.889	0.20457
20.0	50.0	0.848	0.73011
50.0	50.0	0.853	0.70238
100.0	50.0	0.887	0.52165

PD CD34+ AML

ABBV-075 (nM)	ABT-199 (nM)	Effect	CI
50.0	10.0	0.513	0.02929
100.0	10.0	0.523	0.02233
250.0	10.0	0.577	0.00652
50.0	20.0	0.556	0.01074
100.0	20.0	0.526	0.03591
250.0	20.0	0.524	0.04530

Figure S9

PD CD34+ AML

ABBV-075 (nM)	ABT-199 (nM)	Effect	CI
20.0	10.0	0.536	0.69403
50.0	10.0	0.543	0.67240
100.0	10.0	0.548	0.65734
250.0	10.0	0.528	0.71954
50.0	20.0	0.679	0.70350
100.0	20.0	0.713	0.58722
250.0	20.0	0.691	0.66089

PD CD34+ AML

ABBV-075 (nM)	ABT-199 (nM)	Effect	CI
20.0	10.0	0.573	0.00854
50.0	10.0	0.595	0.00607
100.0	10.0	0.611	0.00480
20.0	20.0	0.611	0.00136
50.0	20.0	0.701	9.59 E-5
100.0	20.0	0.684	1.74E-4

PD CD34+ AML

ABBV-075 (nM)	ABT-199 (nM)	Effect	CI
10.0	20.0	0.491	0.03297
20.0	20.0	0.517	0.02904
50.0	20.0	0.543	0.03200
100.0	20.0	0.59	0.02717
250.0	20.0	0.613	0.04146
10.0	50.0	0.539	0.03932
20.0	50.0	0.61	0.01745
50.0	50.0	0.634	0.01590
100.0	50.0	0.66	0.01478
250.0	50.0	0.663	0.02470

PD CD34+ AML

ABBV-075 (nM)	ABT-199 (nM)	Effect	CI
20.0	10.0	0.268	0.38760
50.0	10.0	0.362	0.50415
100.0	10.0	0.473	0.54426
20.0	20.0	0.519	0.08776
50.0	20.0	0.539	0.19357
100.0	20.0	0.605	0.26960

Figure S10

PD CD34+ AML

A-1210477 (µM)	ABBV-075 (nM)	Effect	CI
2.0	20.0	0.3964	0.40621
2.0	50.0	0.4118	0.52400
2.0	100.0	0.4698	0.25387
5.0	20.0	0.5997	0.15391
5.0	50.0	0.5831	0.17009
5.0	100.0	0.6047	0.15184
10.0	20.0	0.8064	0.08141
10.0	50.0	0.8467	0.05646
10.0	100.0	0.8633	0.04746

PD CD34+ AML

A-1210477 (µM)	ABBV-075 (nM)	Effect	CI
2.0	20.0	0.4276	0.03706
2.0	50.0	0.3913	0.23960
2.0	100.0	0.4312	0.04199
5.0	20.0	0.3879	0.23930
5.0	50.0	0.4397	0.07770
5.0	100.0	0.4492	0.06985
10.0	20.0	0.5337	0.05430
10.0	50.0	0.5251	0.05964
10.0	100.0	0.5707	0.03615

PD CD34+ AML

A-1210477 (µM)	ABBV-075 (nM)	Effect	CI
2.0	20.0	0.2377	0.85025
2.0	50.0	0.3132	0.43475
2.0	100.0	0.4103	0.19749
5.0	20.0	0.3409	0.72157
5.0	50.0	0.3539	0.67026
5.0	100.0	0.4678	0.27047
10.0	20.0	0.5552	0.25757
10.0	50.0	0.6390	0.13167
10.0	100.0	0.6327	0.13983

PD CD34+ AML

A-1210477 (µM)	ABBV-075 (nM)	Effect	CI
2.0	20.0	0.3584	0.17587
2.0	50.0	0.360	0.18942
2.0	100.0	0.4049	0.13566
5.0	20.0	0.5279	0.17885
5.0	50.0	0.5098	0.19512
5.0	100.0	0.5285	0.17835
10.0	20.0	0.751	0.10881
10.0	50.0	0.7875	0.08499
10.0	100.0	0.8124	0.07051

Figure S10

PD CD34+ AML

A-1210477 (μM)	ABBV-075 (nM)	Fa	CI Value
2.0	20.0	0.715	0.42908
2.0	50.0	0.763	0.50417
2.0	100.0	0.7822	0.70809
5.0	20.0	0.8225	0.21166
5.0	50.0	0.8446	0.23253
5.0	100.0	0.8215	0.48038
10.0	20.0	0.9234	0.11512
10.0	50.0	0.9177	0.13565
10.0	100.0	0.8948	0.22494

Figure S11

PD CD34+ AML

A-1210477 (μM)	ABT-199 (nM)	Effect	CI
2.0	10.0	0.2857	0.10529
5.0	10.0	0.39	0.01353
10.0	10.0	0.604	3.18E-4
2.0	20.0	0.3532	0.04557
5.0	20.0	0.4189	0.01342
10.0	20.0	0.6136	3.95E-4
2.0	50.0	0.4041	0.03913
5.0	50.0	0.5085	0.00549
10.0	50.0	0.6916	1.56E-4

PD CD34+ AML cells

A-1210477 (μM)	ABT-199 (nM)	Effect	CI
2.0	10.0	0.2094	0.24978
5.0	10.0	0.2941	0.32612
10.0	10.0	0.5213	0.16813
2.0	20.0	0.2286	0.21233
5.0	20.0	0.3562	0.21855
10.0	20.0	0.4917	0.19872
5.0	50.0	0.2701	0.38573
5.0	50.0	0.3619	0.21104
10.0	50.0	0.5201	0.16928

PD CD34+ AML cells

A-1210477 (μM)	ABT-199 (nM)	Effect	CI
2.0	10.0	0.2839	0.08734
5.0	10.0	0.3856	0.00773
10.0	10.0	0.66	1.92E-5
2.0	20.0	0.3683	0.02137
5.0	20.0	0.4418	0.00422
10.0	20.0	0.6568	3.78E-5
2.0	50.0	0.4007	0.02520
5.0	50.0	0.5291	0.00155
10.0	50.0	0.7328	1.28E-5

Figure S12

A

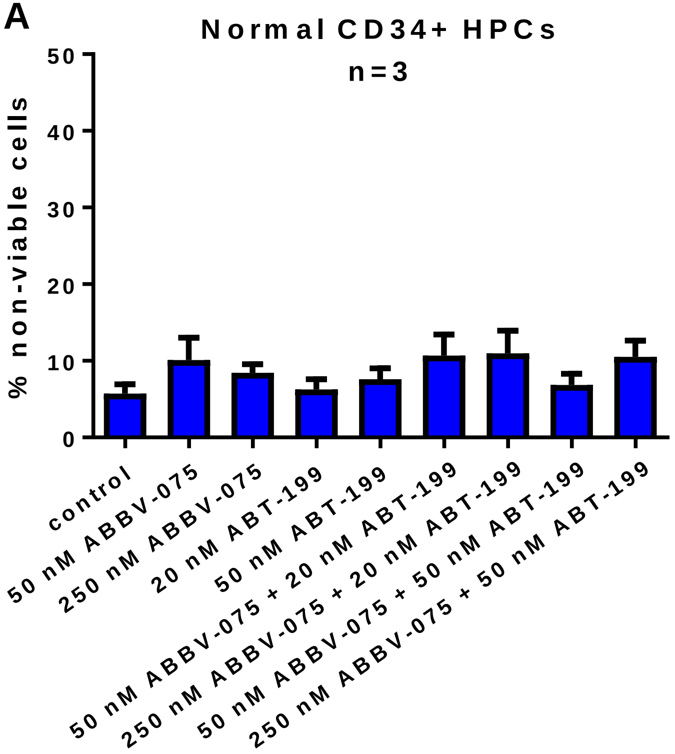

B

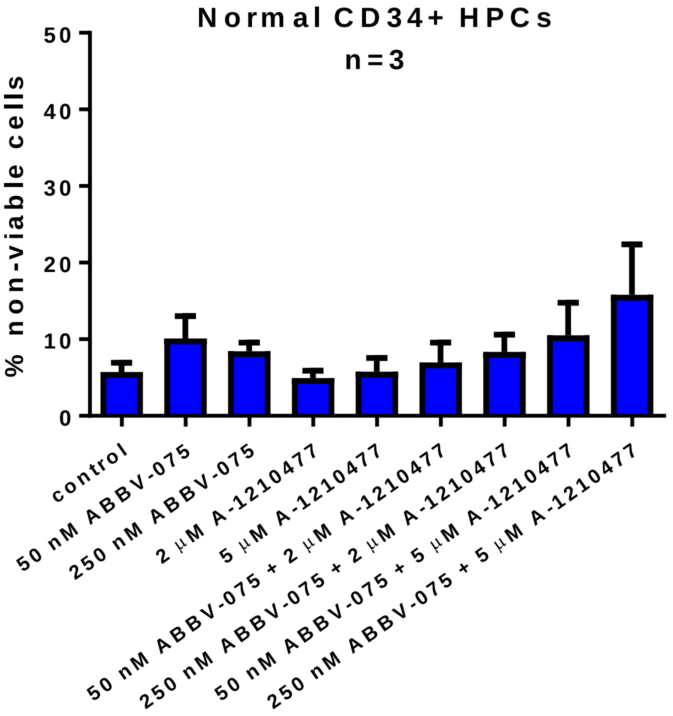

Supplement: Supplementary file 1 — Supplemental Data Figures [file 41408_2018_165_MOESM1_ESM.pdf]
