# Supplementary material for: Superior efficacy of cotreatment with BET protein inhibitor and BCL2 or MCL1 inhibitor against AML blast progenitor cells
Source: Blood Cancer J. 2019 Jan 15;9(2):4. doi: 10.1038/s41408-018-0165-5 (PMC6333829; doi:10.1038/s41408-018-0165-5)
Supplement: Supplementary file 2 — Supplemental Figure Legends [file 41408_2018_165_MOESM2_ESM.docx]

**Supplemental Figure Legends**

**Supplemental Figure 1. Treatment with ABBV075 does not alter the profile of H3K4Me3 or H3K27Ac on the Bcl-xL and MCL1 promoter in AML cells**. **A-D**. OCI-AML5 and MV4-11 cells were treated with 250 nM of ABBV-075 for 8 hours. Chromatin immunoprecipitation was performed with antibodies for H3K4Me3 and H3K27Ac. The resulting ChIP’ed DNA was utilized for qPCR. The presence of each mark on the promoter region of Bcl-xL or MCL1 with and without treatment with ABBV-075 was determined utilizing the Ct value of the ChIP’ed DNA and the Ct value of the input DNA. Abundance of each mark is reported as a % of the input DNA.

**Supplemental Figure 2**. **Treatment with BET inhibitor ABBV-075 depletes BCL2 and induces BIM and p27 in AML cells**. **A**. Oncoplot of common AML-relevant mutations in the AML cell lines utilized in these studies. **B**. MV4-11 cells were treated with 250 nM of ABBV-075 in biologic triplicates for 16 hours. Cells were harvested and RPPA analysis was conducted. The heat map shows the number of proteins down or upregulated proteins greater than 20% relative to control cells and with a p-value < 0.05. **C**. The log2 fold-change of selected up and downregulated proteins following treatment with ABBV-075 compared to control cells. **D**. MOLM13 cells were treated with the indicated concentrations of ABBV-075 for 24 hours. Total cells lysates were prepared and immunoblot analyses were conducted. The expression levels of β-Actin in the cell lysates served as the loading control. The numbers beneath the bands represent densitometry analysis.

**Supplemental Figure 3**. **Treatment with BET inhibitor ABBV-075 depletes MCL1 and induces BIM and p21 in PD-AML BPCs**. **A-B**. Densitometry analysis performed on MCL1 expression in MV4-11 and OCI-AML5 cells treated with ABBV075. * p < 0.05; ** p < 0.01; *** p < 0.005 compared to the untreated control cells. **C-E**. Patient-derived AML blast progenitor cells were treated with the indicated concentrations of ABBV-075 for 24 hours. Total cells lysates were prepared and immunoblot analyses were conducted. The expression levels of β-Actin in the cell lysates served as the loading control. **F**. Densitometry analysis performed on MCL1 expression in PD AML BPCs cells treated with ABBV075. * p < 0.05 compared to the untreated control cells.

**Supplemental Figure 4**. **Treatment with ABBV-075 dose dependently induces apoptosis in genetically diverse subsets of cultured AML cells**. **A**. OCI-AML5 and MOLM13 cells were treated with the indicated concentrations of ABBV-075 for 24 hours. Total cell lysates were prepared and immunoblot analysis was conducted. The expression levels of β-Actin in the lysates served as the loading control. **B**. OCI-AML5 and MOLM13 cells were treated with the indicated concentrations of ABBV-075 for 16 hours. The expression of BAK-NT or BAX6A7 in the cells was determined by flow cytometry. Values shown are fold-change over the untreated control cells. **C-D**. MV4-11, MOLM13, Mono-Mac-1, OCI-AML5, and SKM1 cells were treated with the indicated concentrations of ABBV-075 for 48 hours. At the end of treatment, the % of annexin V-positive, apoptotic cells was determined by flow cytometry. Columns, mean of three experiments; Bars, Standard error of the mean. **E**. PD, CD34+AML cells were treated with the indicated concentrations of ABBV-075 for 48 hours. At the end of treatment, cells were stained with To-Pro-3 iodide and the % of non-viable cells were determined by flow cytometry. A scatter plot was generated utilizing GraphPad V7. **F**. The PD, CD34+ AML cells from panel E, arranged by treatment status (untreated or treatment-refractory). **G**. Oncoplot of mutations in the primary, patient-derived (PD) AML cells utilized in these studies as determined by a 28-gene next generation sequencing panel.

**Supplemental Figure 5**. **Treatment with ABT-199 or MCL1 inhibitor A-1210477 induces MCL1 expression levels, activation of BAK and cleaved PARP in AML cells**. **A**. MOLM13 and OCI-AML5 cells were treated with the indicated concentrations of ABT-199 for 16 hours. Cells were fixed, permeabilized and stained with BAK-NT antibody, then analyzed by flow cytometry. **B**. OCI-AML5 cells were treated with the indicated concentrations of ABT-199 for 24 hours. Total cell lysates were prepared and immunoblot analysis was conducted. The expression levels of β-Actin in the cell lysates served as the loading control. **C**. Densitometry analysis performed on MCL1 expression in MOLM13, MV4-11 and OCI-AML5 cells treated with ABT-199. * p < 0.05; *** p < 0.005. **D-E**. MV4-11 and MOLM13 cells were treated with the indicated concentrations of A-1210477 for 24 hours. Total cell lysates were prepared and immunoblot analyses were conducted. The expression levels of β-Actin in the cell lysates served as the loading control. **F**. MOLM13 and MV4-11 cells were treated with the indicated concentrations of A-1210477 for 16 hours. Cells were stained with anti-BAK-NT antibody and analyzed by flow cytometry. **G**. SKM1 cells were treated with ABBV-075 as indicated for 24 hours. Total cell lysates were prepared and immunoblot analyses were conducted. The expression levels of β-Actin in the cell lysates served as the loading control. **H**. OCI-AML5 cells were treated with the indicated concentrations of A-1210477 for 24 hours. Total cell lysates were prepared and immunoblot analyses were conducted. The expression levels of β-Actin in the cell lysates served as the loading control.

**Supplemental Figure 6**. **Co-treatment with ABBV-075 and ABT-199 exerts synergistic lethal activity against cultured AML cells**. **A-D**. Dose and Fractional effect tables for the associated CI values in MV4-11, MOLM13, OCI-AML5 and SKM1 cells treated with ABBV-075 and ABT-199 for 48 hours.

**Supplemental Figure 7**. **Co-treatment with ABBV-075 and MCL1 inhibitor exerts synergistic lethal activity against cultured AML cells**. **A**. MV4-11 and OCI-AML5 cells were treated with the indicated concentrations of ABBV-075 and/or ABT-199 for 24 hours. Total cell lysates were prepared and immunoblot analyses were conducted. The expression levels of β-Actin in the cell lysates served as the loading control. **B-E**. Dose and Fractional effect tables for the associated CI values in MV4-11, MOLM13, OCI-AML5 and SKM1 cells treated with A1210477 and ABBV-075 for 48 hours **F**. OCI-AML5 cells were treated with the indicated concentrations of ABBV-075 and/or A-1210477 for 24 hours. Total cell lysates were prepared and immunoblot analyses were conducted. The expression levels of β-Actin in the cell lysates served as the loading control.

**Supplemental Figure 8**. **Co-treatment with MCL1 inhibitor A-1210477 and ABT-199 exerts synergistic lethal effects against cultured AML cells**. MOLM13, MV4-11, OCI-AML5 and SKM1 cells were treated with A-1210477 and ABT-199 alone and in combination for 48 hours. Following this, the % of annexin V-positive, apoptotic cells was determined by flow cytometry. **A**. Dose and fractional effect tables for the 4 AML cell lines treated with the combinations. **B**. Combination index values for A-1210477 and ABT-199 were calculated by Compusyn and graphed utilizing GraphPad V7.

**Supplemental Figure 9**. **Co-treatment with ABBV-075 and ABT-199 exerts synergistic lethal activity against PD CD34+ AML BPCs**. Dose and Fractional effect tables for the associated CI values in PD CD34+ AML cells treated with ABBV-075 and ABT-199 for 48 hours.

**Supplemental Figure 10**. **Co-treatment with ABBV-075 and MCL1 inhibitor A-1210477 exerts synergistic lethal activity against PD CD34+ AML BPCs**. Dose and Fractional effect tables for the associated CI values in PD CD34+ AML cells treated with ABBV-075 and A-1210477 for 48 hours.

**Supplemental Figure 11**. **Co-treatment with A-1210477 and ABT-199 exerts synergistic lethal activity against PD CD34+ AML BPCs**. Dose and Fractional effect tables for the associated CI values in PD CD34+ AML cells treated with A-1210477 and ABT-199 for 48 hours.

**Supplemental Figure 12**. **Co-treatment with ABBV-075 and ABT-199 or A-1210477 does not induce significant loss of viability of normal CD34+ hematopoietic progenitor cells. A-B.** Normal CD34+ cord blood progenitor cells (n=3) were treated with the indicated concentrations of ABBV-075 and/or ABT-199 or A-1210477 for 48 hours. At the end of treatment, cells were washed with 1X PBS and stained with To-Pro-3 iodide. The percentage of To-Pro-3 iodide-positive, non-viable cells were analyzed by flow cytometry.
