# Supplementary material for: Superior efficacy of cotreatment with BET protein inhibitor and BCL2 or MCL1 inhibitor against AML blast progenitor cells
Source: Blood Cancer J. 2019 Jan 15;9(2):4. doi: 10.1038/s41408-018-0165-5 (PMC6333829; doi:10.1038/s41408-018-0165-5)
Supplement: Supplementary file 3 — Extended and Supplemental Methods [file 41408_2018_165_MOESM3_ESM.docx]

**Supplemental Methods**

**Sequencing of primary AML cells.** We performed targeted next-generation sequencing (NGS) of DNA samples from bone marrow or peripheral blood collected from patients at our center with AML as described (1). Diagnostic bone marrow samples were obtained for mutational analysis. Total genomic DNA was extracted from unenriched peripheral blood (PB) or bone marrow (BM) samples using ReliaPrep genomic DNA isolation kit (Promega Corp, Madison, WI, USA). FLT3 (internal tandem duplication and D835) was assessed by PCR followed by capillary electrophoresis on a Genetic Analyzer (Applied Biosystems, Foster City, CA, USA). Briefly, a total of 250 ng DNA was utilized to prepare sequencing libraries using Agilent HaloPlex custom Kit (Agilent Technologies, Santa Clara, CA, USA). The entire coding sequences of 28 genes (ABL1, ASXL1, BRAF, DNMT3A, EZH2, FLT3, GATA1, GATA2, HRAS, IDH1, IDH2, IKZF2, JAK2, KIT, KRAS, MDM2, MLL, MPL, NPM1, NRAS, PTPN11, RUNX1, TET2, TP53, WT1) were interrogated on a custom-designed next-generation sequencing approach using the Illumina MiSeq platform (Illumina; San Diego, CA, USA). The genomic reference sequence used was genome GRch37/hg19. The following software tools were utilized in the experimental setup and data analysis: Illumina Experiment Manager 1.6.0 (Illumina; San Diego, CA, USA), MiSeq Control Software 2.4 (Illumina; San Diego, CA, USA), Real Time Analysis 1.18.54 (Illumina; San Diego, CA, USA), Sequence Analysis Viewer 1.8.37 (Illumina; San Diego, CA, USA), MiSeq Reporter 2.5.1 (Illumina; San Diego, CA, USA), and SureCall 3.0.1.4 (Agilent Technologies; Santa Clara, CA, USA). A minimum of 80% reads at quality scores of AQ30 or higher were required to pass quality control.

**Analysis of epigenetic state in AML cells *in vitro***. ATAC-Seq analysis of AML SET2 cells with and without treatment with BET inhibitor OTX015 was performed following a previously described protocol (2) with modifications_._ Libraries were generated with a Nextera DNA Library Preparation Kit containing the mutant Tn5 transposase (Illumina, San Diego, CA; Catalog number: FC-121-1030). The DNA fragments were indexed utilizing a Nextera Index Kit (Illumina, San Diego, CA; Catalog number: FC-121-1011) and amplified by PCR according to the manufacturer’s protocol. The amplified library fragments were PCR purified with a Qiagen Min Elute column, pooled and sequenced on a HiSeq4000 next generation sequencer. The ATAC-Seq reads were processed and Log2 fold-changes were calculated utilizing diffReps (3). Transcription Factor binding motifs in the ATAC-Seq peak data were determined with HOMER (4).

**Cell lysis and protein quantitation.** Untreated or drug-treated cells were centrifuged, and the cell pellets were incubated in lysis buffer on ice for 30 minutes, as previously described (5,6). After centrifugation, an aliquot of each cell lysate was diluted 1:10 and the protein content was quantitated using a BCA protein quantitation kit (Pierce, Rockford, IL), according to the manufacturer’s protocol. The protein concentrations were determined against a standard curve of BSA measured in a BioTek Synergy H1 plate reader (Winooski, VT) at 562 nm wavelength. Samples were prepared in SDS sample buffer and boiled for 5 minutes prior to loading.

**SDS-PAGE and immunoblot analyses.** Seventy five micrograms of total cell lysate were used for SDS-PAGE. Western blot analyses were performed on total cell lysates using specific antisera or monoclonal antibodies. Blots were washed with 1X PBST, then incubated in IRDye 680 goat anti-mouse or IRDye 800 goat anti-rabbit secondary antibodies (LI-COR, Lincoln, NE) for 1 hour, washed 3 times in 1X PBST and scanned with an Odyssey CLX Infrared Imaging System (LI-COR, Lincoln, NE). The expression levels of β-Actin in the cell lysates were used as the loading control for the Western blots. Immunoblot analyses were performed at least twice. Representative immunoblots were subjected to densitometry analysis. Densitometry was performed using ImageJ software (7). Values were graphed in GraphPad V7 (La Jolla, CA).

**Reverse phase protein array analysis.** RPPA was performed in the Functional Proteomics RPPA core facility at the MD Anderson Cancer Center. This array allows the simultaneous detection of 304 unique antibodies against human proteins. This array is curated and highly validated. Briefly, cell lysates were serially diluted two-fold for 5 dilutions (from undiluted to 1:16 dilution) and arrayed on nitrocellulose-coated slides in an 11 x 11 format. Samples were probed with antibodies by tyramide-based signal amplification approach and visualized by DAB colorimetric reaction. Slides were scanned on a flatbed scanner to produce 16-bit tiff image. Spots from tiff images were identified and the density was quantified by Array-Pro Analyzer. Relative protein expression for each sample were normalized by interpolation of each dilution curves from the "standard curve" (supercurve) of the slide (antibody). Supercurve is constructed by a script in R, written by the Bioinformatics Department at the University of Texas MD Anderson Cancer Center (8). These values (given as Log2 values) are defined as Supercurve Log2 (Raw) values and imported into an Excel worksheet. All the data points were normalized for protein loading and transformed to linear value, designated as "Normalized Linear" (labeled "NormLinear" in the worksheet). "Normalized Linear" values were transformed to Log2 values (labeled "NormLog2" in worksheet), and then median-centered for hierarchical clustering analysis (labeled "NormLog2_MedianCentered" in the worksheet). Median-centered values were then formatted for heatmap generation in the "Format for Heatmap" worksheet. Our data were further processed and our heatmaps display only proteins that were altered greater than or equal to 20% up or down and had a p-value of less than 0.05. Multiple hypotheses testing correction was applied using the false discovery rate (fdr) method as implemented in the R statistical system.

**Analysis of BAX and BAK conformation change.** After the designated treatments, cells were harvested by centrifuging at 125 x g for 5 minutes. Cells were washed once with 1× phosphate-buffered saline (PBS) in 12 x 75 mm flow tubes, resuspended in 100 µL of 4% para-formaldehyde/PBS (prepared from 16% EM grade methanol free-paraformaldehyde [Electron Microscopy Sciences, Hatfield, PA]) then vortexed to mix, and incubated at 37°C for 10 minutes. Nine hundred microliters of 100% methanol were added and the tubes were incubated for 2 hours to overnight at -20°C. Fixed cells were washed twice with 1× PBS by centrifuging at 125 x g for 5 minutes and then stained in 100 µL of antibody staining buffer (0.5% BSA/PBS) with Alexa Fluor 488-conjugated anti-BAX6A7 or with anti-BAK(NT) antibody. For BAX6A7 detection, cells were washed with 0.5% BSA/PBS, re-suspended in 0.5% BSA/PBS and kept on ice in the dark for flow cytometry analysis. For BAK-(NT) staining, cells were washed with 0.5% BSA/PBS then counterstained with Alexa Fluor 488-conjugated anti-rabbit secondary antibody (in 0.5% BSA/PBS) in the dark, on ice for 1 hour. Cells were washed with 0.5% BSA/PBS, re-suspended in 0.5% BSA/PBS and kept on ice for flow cytometry analysis. Data were collected on a flow cytometer with a 488 nM laser in the FL1 channel and analyzed with Accuri CFlow6 software (BD Biosciences).

**Assessment of apoptosis by annexin-V staining.** Untreated or drug-treated cells were stained with Annexin-V (Pharmingen, San Diego, CA) and TO-PRO-3 iodide (Life Technologies, Carlsbad, CA) and the percentages of apoptotic cells were determined by flow cytometry. To analyze synergism between ABBV-075 and ABT-199 or A-1210477 or synergy between A1210477 and ABT-199, cells were treated with single agents and combinations for 48 hours and the percentages of annexin V-positive, apoptotic cells were determined by flow cytometry. The combination index (CI) for each drug combination was calculated by median dose effect and isobologram analyses (assuming mutual exclusivity) utilizing the commercially available software Compusyn (9). CI values of less than 1.0 represent a synergistic interaction of the two drugs in the combination. The CI values were input into GraphPad V7.0 to create the Box and Whisker plots of the range of the CI values for each cell line and combination.

**Chromatin immunoprecipitation and Real Time Polymerase Chain Reaction.** OCI-AML5 or MV4-11 cells were treated with ABBV-075 for 8 hours. Following drug exposure, chromatin was cross-linked with methanol-free formaldehyde at a final concentration of 1% and incubated at room temperature for 10 minutes. Glycine was utilized to quench the crosslinking reaction. Cells were washed with 1X PBS and centrifuged for 5 minutes at 2000 rpm. The cell pellets were snap-frozen in liquid nitrogen. Cell lysis, sonication and chromatin immunoprecipitation for H3K4Me3 and H3K27Ac was performed according to the manufacturer’s protocol (Millipore). Immunoprecipitated DNA was treated with Proteinase K for 1 hour. DNA crosslinks were reversed by incubation at 65°C for a minimum of 4 hours in a thermocycler. DNA was purified utilizing a PCR purification kit and eluted in 50 microliters. For quantitative assessment of binding of H3K4Me3 and H3K27Ac to the Bcl-xL and MCL1 promoter in the chromatin immunoprecipitates, a SYBR Green PCR Mastermix from Applied Biosystems was used (Foster City, CA). Primers utilized for assessment of binding to Bcl-xL and MCL-1 promoters were Bcl-xL Prom For 1 (5’-CCTACTGGGAGCCAGGAGTA-3’), Bcl-xL Prom Rev 1 (5’-CCTGGGCTGGTGCTTAAATA-3’), Bcl-xL Prom For 2 (5’-GCAATCTGACTTTGGGAAGG-3’), Bcl-xL Prom Rev 2 (5’-TTGGGGAATTCAGAGCAAAC-3’), MCL1 Prom For 1 (5’-CCCCCACAGTAGAGGTTGAG-3’), MCL1 Prom Rev 1 (5’-GGGTCTTCCCCAGTTTTCTC-3’), MCL1 Prom For 3 (5’-GCTCCACGTGCTACCCTAAA-3’) and MCL1 Prom Rev 3 (5’-AGAGATGGGAGAAGCAAGCA-3). Relative enrichment of the DNA in the chromatin immunoprecipitates was normalized against the amount of Bcl-xL and MCL1 promoter DNA in the input samples.

**REFERENCES for Supplemental Methods**

1. Khan M, Cortes J, Kadia T, Naqvi K, Brandt M, Pierce S, et al. Clinical Outcomes and Co-Occurring Mutations in Patients with RUNX1-Mutated Acute Myeloid Leukemia. Int J Mol Sci 2017; 18: pii: E1618.
2. Buenrostro JD, Wu B, Chang HY, Greenleaf WJ. ATAC-seq: A Method for assaying chromatin accessibility genome-wide. Curr Protoc Mol Biol 2015; 109, 21.29.1-9.
3. Shen L, Shao N-Y, Liu X, Maze I, Feng J, Nestler EJ. diffReps: Detecting Differential Chromatin Modification Sites from ChIP-seq Data with Biological Replicates. PLoS ONE. 2013; 8: e65598. doi:10.1371/journal.pone.0065598.
4. Heinz S, Benner C, Spann N, Bertolino E, Lin YC, Laslo P et al. Simple Combinations of Lineage-Determining Transcription Factors Prime cis-Regulatory Elements Required for Macrophage and B Cell Identities. Mol Cell 2010; 38: 576-589.
5. Wang Y, Fiskus W, Chong DG, Buckley KM, Natarajan K, Rao R, et al. Cotreatment with panobinostat and JAK2 inhibitor TG101209 attenuates JAK2V617F levels and signaling and exerts synergistic cytotoxic effects against human myeloproliferative neoplastic cells. Blood 2009; 114: 5024-5033.
6. Fiskus W, Verstovsek S, Manshouri T, Rao R, Balusu R, Venkannagari S, et al. Heat Shock Protein 90 Inhibitor Is Synergistic with JAK2 Inhibitor and Overcomes Resistance to JAK2-TKI in Human Myeloproliferative Neoplasm Cells. Clin Cancer Res 2011; 17: 7347-7358.
7. Schneider CA, Rasband WS, Eliceiri KW. NIH Image to ImageJ: 25 years of image analysis. Nat Methods 2012; 9: 671-675.
8. Troncale S, Barbet A, Coulibaly L, Henry E, He B, Barillot E, et al. NormaCurve: a SuperCurve-based method that simultaneously quantifies and normalizes reverse phase protein array data. PLoS One 2012; 7: e38686.
9. Chou TC, Talalay P. Quantitative analysis of dose-effect relationships: the combined effects of multiple drugs or enzyme inhibitors. Adv Enzyme Regul 1984; 22: 27-55.
